# A Brief Review of H_2_S and Nitrogen-Based Contaminants from Biogas: Effects on Reforming Catalysts and SOFC Anodes

**DOI:** 10.1021/acsomega.5c02399

**Published:** 2025-11-18

**Authors:** Daiana Gotardo Martinez, Ligia Gomes Oliveira, Matheus Henrique Zanardini, Matheus Ribeiro de Jesus Cerqueira, João Pedro Jenson de Oliveira, Fabio Coutinho Antunes, Gustavo Doubek, Hudson Zanin, Julian Hunt

**Affiliations:** † 696466 International Center for Renewable Energy and Biogas (CIBiogás), Itaipu Technological Park, St. Presidente Tancredo Neves, 6731, 85867-900 Foz Do Iguaçu, Parana, Brazil; ‡ Renewable Materials and Energy Laboratory (LABMATER), Federal University of Parana (UFPR - Setor Palotina), St. Pioneiro, 2153, 85950-000 Palotina, Parana, Brazil; § School of Electrical and Computer Engineering, University of Campinas, Av. Albert Einstein 400, 13083-852 Campinas, SP, Brazil; ∥ Centre for Energy and Oil Studies, University of Campinas, Av. Cora Coralina 350, 13083-896 Campinas, SP, Brazil; ⊥ School of Chemical Engineering, 529941 University of Campinas, Av. Albert Einstein 500, 13083-852 Campinas, SP, Brazil; # Biological and Environmental Science and Engineering Division, 564519 King Abdullah University of Science and Technology, Thuwal 23955-6900, Saudi Arabia

## Abstract

This paper provides a brief review of biogas contaminants, examining the impact of hydrogen sulfide (H_2_S), nitrogen (N_2_), and ammonia (NH_3_) on catalytic reforming processes and Solid Oxide Fuel Cells (SOFCs) electricity generation. Biogas, produced through anaerobic digestion (AD), is a promising renewable energy source that can be upgraded into biomethane and reformed into H_2_-rich syngas for SOFCs. Despite this, contaminants like H_2_S found in biogas and biomethane cause critical issues such as catalyst poisoning, decreased electrochemical performance, and higher maintenance costs, while NH_3_, potentially formed from N_2_ during reforming, can lead to catalyst degradation and reduced SOFC performance, representing an underexplored contamination pathway. This review highlights the negative impacts of H_2_S, N_2_, and NH_3_ on catalytic reforming and SOFCs, causing catalyst deactivation and performance loss, thus reinforcing the importance of developing advanced purification methods and sulfur-tolerant materials to improve renewable hydrogen (H_2_) production and fuel cell durability.

## Graphical abstract

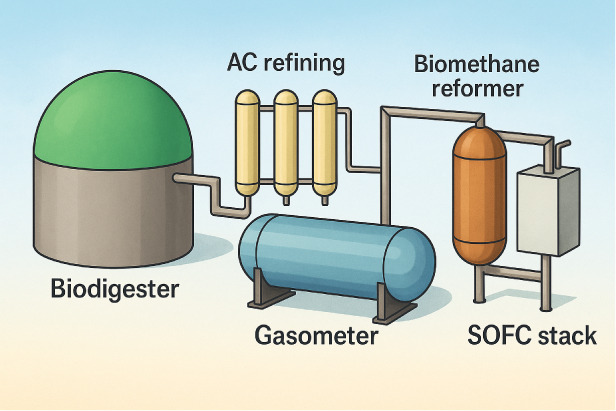

## 1 Introduction

The increasing global demand for sustainable energy has directed significant attention toward renewable sources. Biogas and biomethane production have emerged as a promising environmental solution. Derived from organic waste substrates through AD, biogas and biomethane not only contribute to waste management but also offer a viable alternative to fossil fuels. The transition from conventional to renewable energy sources is a critical step toward mitigating environmental impacts and promoting a sustainable energy future.^1^ Biogas and biomethane are valued for their role in renewable energy generation and as potential feedstock for syngas (H_2_ and carbon monoxide (CO)) produced by catalytic reforming, where H_2_ is recognized for its clean energy potential, especially in fuel cells (FCs), which is critical in transitioning to a low-carbon economy.

FCs differ according to the type of electrolyte and operating conditions, such as (i) Proton Exchange Membrane Fuel Cells (PEMFCs); (ii) Alkaline Fuel Cells (AFCs); (iii) Phosphoric Acid Fuel Cells (PAFCs); (iv) Molten Carbonate Fuel Cells (MCFCs); (v) SOFCs; and (vi) Direct Methanol Fuel Cells (DMFCs). The SOFC stands out due to its high efficiency and fuel flexibility, including H_2_, biomethane, and bioethanol.^2–5^ The utilization of H_2_ in SOFCs enables efficient energy generation with reduced greenhouse gas emissions (GHGs), making it a pivotal technology in the renewable energy landscape.^6^ In addition to their environmental benefits, SOFCs are recognized for their quiet operation, absence of moving parts, high reliability, and superior energy conversion efficiency.^7^

The presence of contaminants in biogas, such as H_2_S, poses significant challenges for syngas production to feed SOFCs. In this scenario, biological desulfurization offers an effective approach to reducing H_2_S levels, although this process also increases N_2_ content. H_2_S is a common impurity in biogas that introduces significant operational challenges in both catalytic reforming processes and SOFCs energy generation. The presence of H_2_S can lead to catalyst poisoning, reduced efficiency, and increased maintenance costs. Conversely, N_2_, which can also be present in syngas obtained from biogas and/or biomethane, has been found to have a positive impact by promoting better H_2_ mass transport inside the anode of SOFCs, thereby enhancing its performance. Understanding the effects of these contaminants is crucial for optimizing the production and utilization of syngas/H_2_ for highly efficient electric power generation.^8^

This study aims to investigate biological desulfurization via in situ microaeration as an approach to biogas desulfurization. The effects of syngas contaminants, particularly H_2_S and N_2_ compounds, on catalytic reforming processes and SOFC performance are also discussed, including the potential formation of NH_3_ from N_2_ under certain conditions, which remains a subject of investigation. By investigating the impact of these impurities, this research seeks to provide insights into improving the efficiency and longevity of renewable H_2_ production and conversion devices and systems. The findings are expected to contribute to developing more robust and efficient renewable energy technologies, supporting the broader goal of a sustainable energy future.

To summarize the scope of this study and provide a conceptual framework for the subsequent analysis, Figure 1 outlines the key topics examined regarding the influence of gas contaminants on catalytic reforming processes and fuel cell-based energy systems

**Figure 1:**
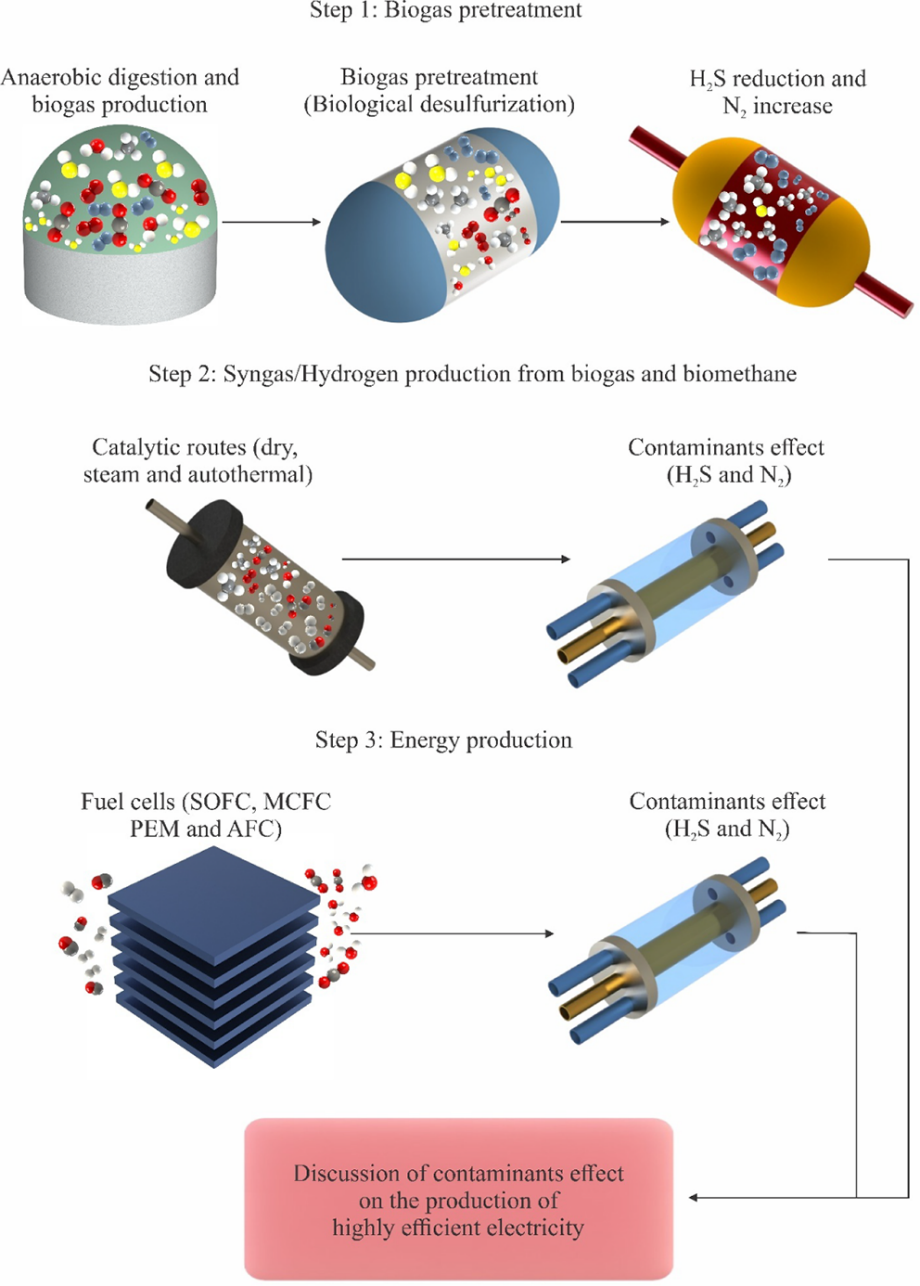
Main summary of the scope of the study on the influence of gaseous contaminants on catalytic reforming and FCs.

## 2 Contaminants in Biogas: Processes for Purification, Including Desulfurization and Nitrogen Control

Carbon dioxide (CO_2_) and biogas contaminants (e.g., H_2_S, NH_3_, N_2_, H_2_) reduce the required properties and the economic value of biogas.^8–11^ These impurities have several detrimental effects, some of them are (i) H_2_S in biogas reacts with metal components in boilers and internal combustion engines, causing corrosion; (ii) silicon compounds deposit on engine surfaces, leading to deterioration of the catalytic converter and exhaust pipes; and (iii) N_2_ and halogenated hydrocarbons compounds negatively affect biogas ignition properties and could trigger engine corrosion in vehicular applications.^12^ For these reasons, pretreating biogas to remove impurities and improve quality is essential. This process enhances equipment lifespan, increases energy efficiency (whether for electrical, thermal, or mobility applications prior to purification), and reduces GHG emissions.^13^ Common biogas pretreatment techniques include desulfurization, absorption, adsorption, and condensation.^14^ The main characteristics of these technologies are summarized in Figure 2.

**Figure 2:**
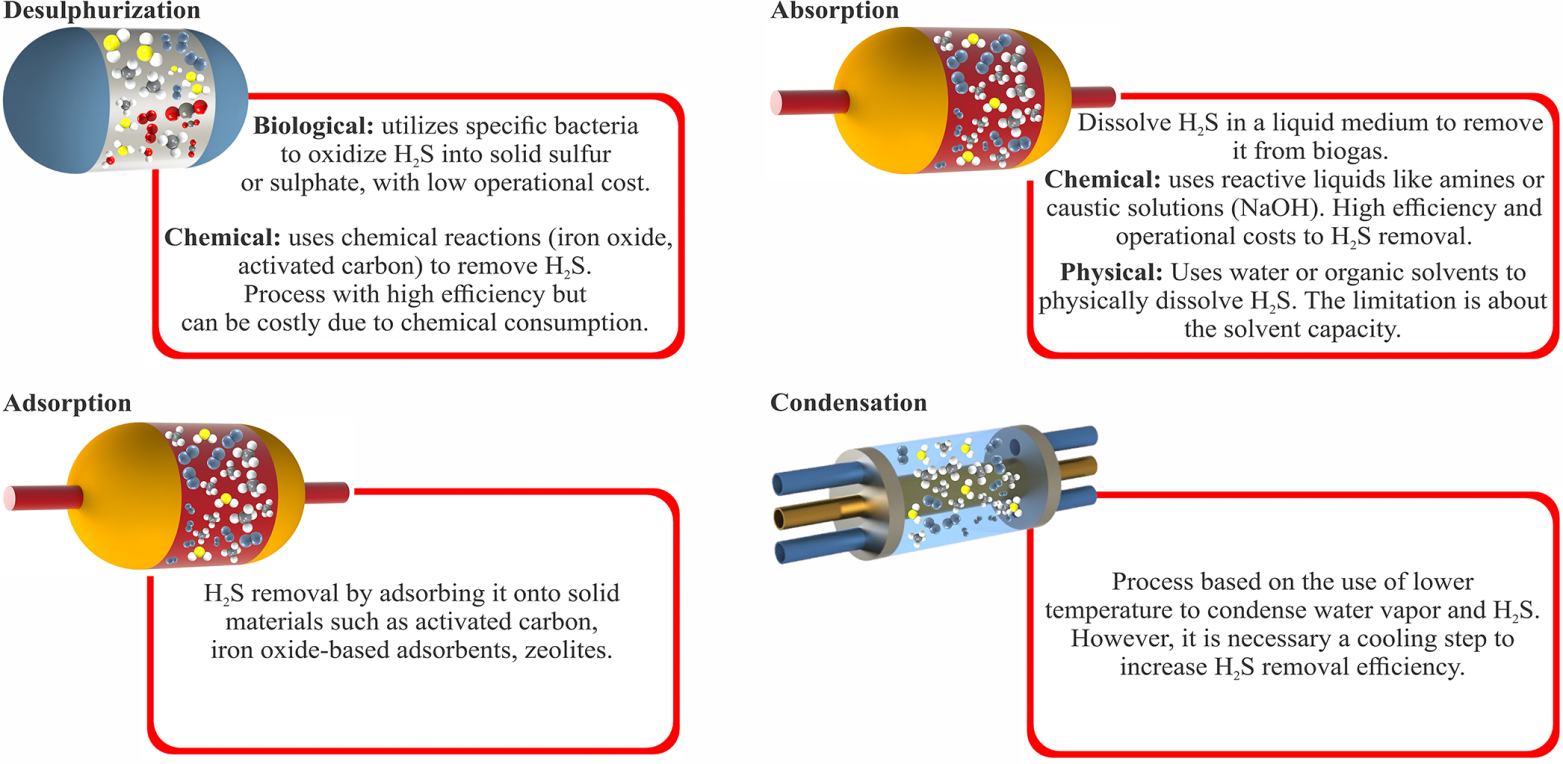
Main processes and technological characteristics for H_2_S removal from biogas.

Along with the contaminants in biogas, H_2_S poses a complete set of challenges, ranging from its corrosive nature to its detrimental effects on health and the environment.^15^ The H_2_S contained in biogas can have a significant impact during its use in energy generation, conversion, and/or synthesis gas production, a technology currently being utilized to harness and valorize biogas. Given those challenges, studies are conducted for the H_2_S removal, with the technologies addressed among the many used for removing this contaminant. It is noteworthy that for each technology, there is a need to assess operational costs, such as the acquisition of systems, chemical reagents, material regeneration, and energy expenses are determinants for the choice of the operating system. Table S1 presents alternative technological pathways for H_2_S removal from biogas. These include biological processes, such as biological desulfurization (BD), and chemical and physical methods (e.g., adsorption and filtration), which are applied postdigestion. Furthermore, each approach exhibits distinct advantages, shortcomings, and challenges, and the selection of the optimal technology depends on process requirements, operational conditions, associated costs, and the initial H_2_S concentration.^16^ Biological desulfurization is often preferred over chemical and physical treatments due to its environmental sustainability, cost-effectiveness,^17^ and high H_2_S removal efficiency without generating harmful byproducts.

Biological technologies, such as the BBC and the BTF, exhibit high efficiency between 88–100%,^18,19^ combined with lower operational costs and low energy demand.^20–22^ This process mainly employs microorganisms such as sulfur-oxidizing bacteria.^23,24^ However, its efficiency depends on strict operational conditions, including pH control, nutrient supply, and proper management of liquid effluents. On the other hand, chemical absorption processes, such as the use of Fe-EDTA-carbonate, demonstrate high efficiency ranging from 96.8% to 100%,^25^ with the additional advantage of enabling the simultaneous removal of other gases such as CO_2_ and oxygen (O_2_),^25,26^ thereby methane (CH_4_) enrichment in the biogas. The Fe^3+^-EDTA complex allows cost-effective oxidation of H_2_S into elemental sulfur (S^0^), and the resulting Fe^2+^-EDTA oxidation can be regenerated back to its active ferric form (Fe^3+^-EDTA) through oxidation with air used in the CO_2_ removal process.^25,27^ Nevertheless, these processes require continuous investment in chemical reagents and the implementation of regeneration systems, which can increase operational costs over time.

In the removal process through membrane-based technologies, such as hollow fiber and polymeric membranes^28,29^ stand out in gas separation processes, particularly for H_2_S removal, due to their high efficiency, modularity, and lower energy consumption compared to conventional processes.^30^ This technology enables the selective removal of H_2_S based on differences in solubility and diffusivity among gas components; however, the choice of membrane material is crucial to ensure chemical resistance against the H_2_S acidity.^31–33^ In addition to H_2_S removal, this process is also effective in separating NH_3_ and CO_2_.^28,34,35^

Specifically, H_2_S removal pathways based on adsorption processes rely on adsorbent materials with specific properties, such as high surface area, porosity, and adsorption capacity. Adsorbents such as biochar,^36^ metal-impregnated activated carbon (Cu/AC, ZnO–CuO/AC),^37,38^ zeolites, and advanced materials like TiO_2_/zeolite and AgNaA nanozeolite^39–42^ exhibit high efficiency in H_2_S removal, with the regeneration capacity of these materials being a key factor in reducing operational costs and enhancing process sustainability.^43^ However, a challenge associated with this technology is the use of silver-based materials, which can significantly increase adsorbent costs.^44^ Within this context, and considering the alternatives discussed above, in situ biological desulfurization, also known as microaeration, emerges as a particularly promising solution due to its high removal efficiency (>99%),^45^ low cost, and minimal impact on the AD process.^46–49^

Microaeration is an in situ technique for biological desulfurization that occurs directly within the liquid phase or in the headspace of the reactor, without the need to add an auxiliary treatment unit. This process is conducted by inserting controlled volumes of air or O_2_ into the reactors (dissolved O_2_ range of 0.1–1.0 mg/L),^50^ favoring the sulfur-oxidizing bacteria (SOBs) such as *Thiobacillus*, *Halothiobacillus*, *Sulfuricurvum*, and *Acinetobacter*.^51^ Figure 3 depicts the schematic representation of biological desulfurization with O_2_ supply. Eqs 1–3 show the sulfide oxidation process using microaeration with O_2_.^49^

**Figure 3:**
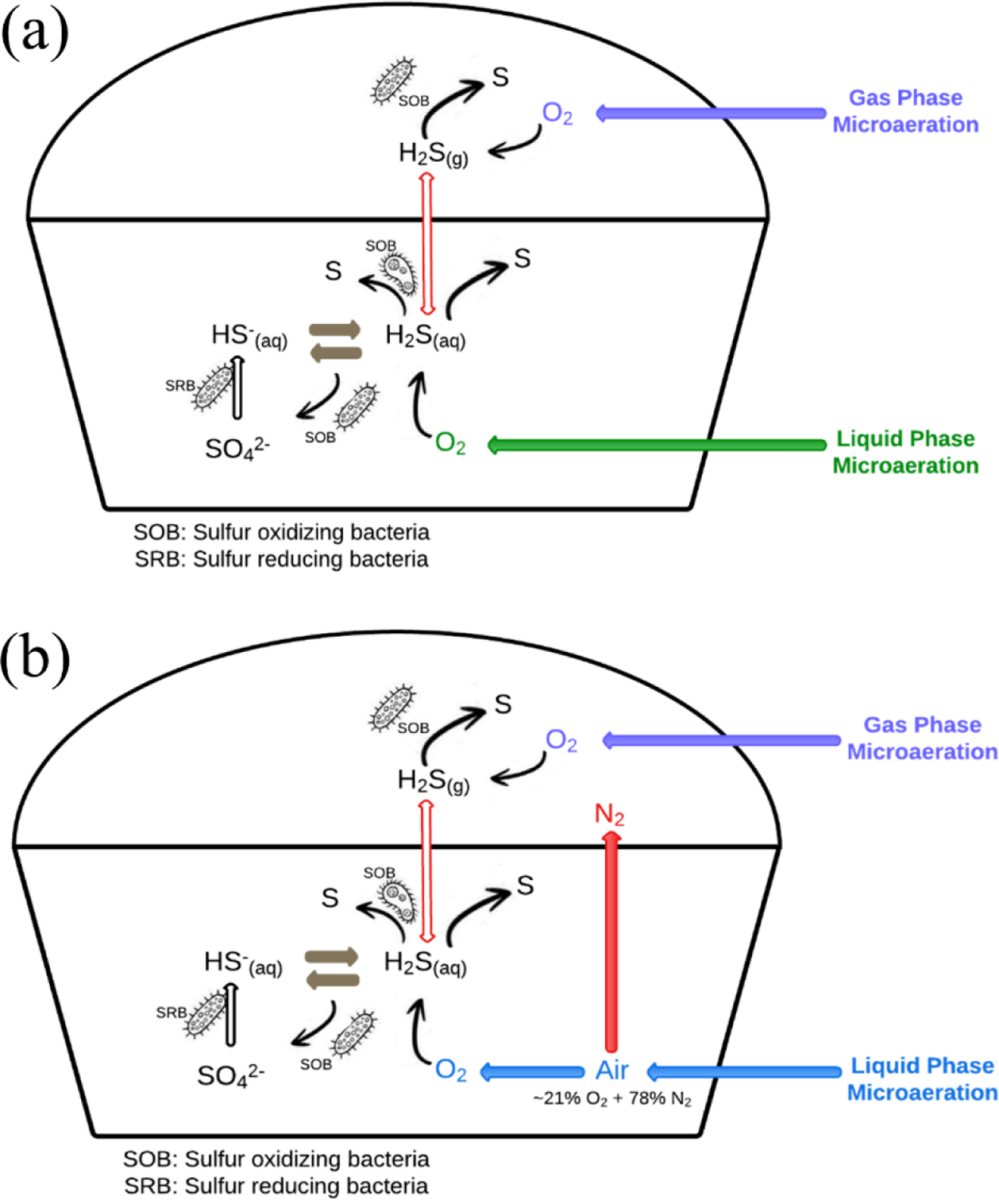
Schematic representation of biological desulfurization with (a) O_2_ and (b) air supply. Adapted with permission from Azizi et al.^52^ Copyright 2022 Published by Elsevier Ltd.


1 $$\begin{array}{l} 2HS^{-}+O_{2}\to 2S^{0}+2OH^{-}\\ \Delta G^{0}=-169.4kJ/mol \end{array}$$

2 $$\begin{array}{l} 2HS^{-}+{4O}_{2}\to 2{{SO}_{4}}^{2-}+2H^{+}\\ \Delta G^{0}=-732.6\;kJ/mol \end{array}$$

3 $$\begin{array}{l} 2HS^{-}+2O_{2}\to S_{2}{O_{3}}^{2-}+H_{2}O\\ \Delta G^{0}=-387.4\;kJ/mol \end{array}$$

The majority of products synthesized from biological desulfurization are elemental sulfur (S^0^) (eq 1) and/or sulfate $\left(SO_{4}^{2-}\right)$ (eq 2). In addition to these, thiosulfate $\left(S_{2}O_{3}^{2-}\right)$ may also be formed (eq 3).^52^ The amount of O_2_ added to the reactor will influence the biological desulfurization reactions. The formation of elemental sulfur requires a lower amount of O_2_ than the formation of sulfates.^49^ The air/O_2_ input into the system will vary according to the H_2_S content produced in the biogas, depending on the type of substrate. Microaeration has a high H_2_S removal efficiency, which can reach values above 99%, as shown in Table S2.

Microaeration is a technology that has already been validated on a full scale. Jenícek et al.^48^ analyzed the performance of microaeration in seven municipal wastewater treatment plants in Central Europe in different periods between 2003 and 2015. The authors reported H_2_S removal efficiencies between 73.8% and 99.5%. In addition, the researchers identified that the presence of N_2_ in the insertion of air into the reactors does not significantly impact the CH_4_ dilution in the system, in some cases reducing the CH_4_ concentration by a maximum of 2%.

Costanzo et al.^8^ reported the efficiency of H_2_S removal using different doses of micro-oxygenation in three thermophilic reactors in series. The high-solid feedstock used was mainly composed of sewage sludge. O_2_ was inserted into the gas phase of the reactors, with a purity of 93 ± 3%, replacing the air insertion that was previously carried out. O_2_ was produced using a pressure-swing adsorption (PSA) system, providing a maximum flow rate up to 3.5 N m^3^/h. Costanzo et al.^8^ achieved a higher H_2_S removal efficiency (98.2%) using a dose of 0.96 ± 0.03 NL O_2_/Nm^3^ biogas. In addition, the authors reported that varying the O_2_ flow rate had no negative impact on AD.

Despite the high efficiency of H_2_S removal, the technique requires some attention. Some limitations of biological desulfurization include the accumulation of elemental sulfur within biodigesters and the potential inhibition of anaerobic microorganisms in AD processes if the airflow into the system becomes unbalanced.^28^ Thus, determining the optimal air injection is crucial to maintaining the high yield of the system, in terms of H_2_S reduction and CH_4_ production. According to Kraakman et al.,^53^ for biogas composition up to 3300 ppm of H_2_S, there is a relation of 1% (v/v) of pure O_2_ or 1 to 8% of air per volume of biogas (1% O_2_ or air/Nm^3^ of biogas). These concentrations mean around 0.1 and 1.75 L/h per m^3^ of the digester, or around 2.5 times the stoichiometric amount of air required for the H_2_S oxidation to elemental sulfur.

Díaz et al.,^54^ investigated the removal of H_2_S via microaerobic processes employing various oxidizing agents, including pure O_2_, air, and nitrate. The study demonstrated that both O_2_ and air achieved up to 99% efficiency in H_2_S removal; however, the use of air resulted in reduced CH_4_ production due to biogas dilution by N_2_. In contrast, nitrate exhibited limited efficacy for H_2_S removal and led to an increase in N_2_ concentration within the biogas. This phenomenon is attributed to heterotrophic denitrification, which competes with methanogenesis, consequently diminishing CH_4_ yields this occurs as denitrifying bacteria prevail, while methanogens are nearly completely inhibited.^55^

Studies indicate that H_2_S removal technology by microaeration has potential as an economical alternative in the biogas desulfurization and purification, as it can be carried out within the anaerobic reactor without the need to build a new separation unit.^47,48^ The free space in the biodigester facilitates biological desulfurization, eliminating the need for CAPEX and OPEX with additional construction, chemicals and energy demand.^56,57^ Nonetheless, there are still knowledge gaps related to the sulfur oxidation (SOM) kinetics and the effects of microaeration on AD performance parameters and the microbial community involved.^58^

Although biogas desulfurization via biological microaeration presents certain challenges, this technology remains a promising approach for developing sustainable and economically viable biogas production pathways with reduced pollutant emissions. Additionally, it enables H_2_S removal before leaving the biodigester, promoting technical safety for equipment and piping, and increasing the CH_4_ content within the system. This technique allows the H_2_S removal by controlled oxidative route using autotrophic microorganisms under mild conditions, which favors the reduction of H_2_S negative impacts over catalyst’s and SOFC anodes performance and lifespan, mainly preventing surface poisoning and materials deactivation, positively influencing the reduction of operating costs.

## 3 Syngas/Hydrogen Production from Biogas and Biomethane

Syngas is a gas mixture comprising H_2_ and CO primarily obtained through CH_4_ reforming^59,60^ and gasification.^61^ It can be a feedstock for renewable hydrocarbons production via Fischer–Tropsch process,^62^ and can also be used for electricity generation in FCs like SOFCs.^63^ Obtaining syngas from biomethane is a promising way to reduce GHGs significantly,^64^ as biomethane can originate from the AD of high organic load waste.^65^ Among the options for obtaining syngas through CH_4_ reforming processes is dry reforming (DRM). This route is particularly interesting because it utilizes CH_4_ and CO_2_ (eq 4).^66^ Autothermal reforming (ATR) is a syngas production process that combines the partial oxidation and steam reforming of CH_4_ (eq 5).^67^ Steam reforming (SRM), the most common industrial route, involves the reaction of CH_4_ with steam (H_2_O vapor) (eq 6) to produce syngas and is well established in natural gas plants.^68,69^ All the aforementioned processes occur in the presence of a heterogeneous catalyst at temperatures above 700 °C.^69,70^ CH_4_ reforming processes vary in catalyst type, operating conditions, and efficiencies (Table S3), with Ni-based catalysts widely applied in DRM and SRM for their high activity and low cost.^67^ Pt and noble metal catalysts (Pd, Rh, Ru) offer high efficiency but are limited by high cost, while perovskite-based materials (e.g La_0.95_Ce_0.05_Ni_0.2_Fe_0.8_O_3_) shows excellent coke resistance and tunable properties,^71–73^ representing promising alternatives.

4 $$\begin{array}{l} CH_{4}+CO_{2}⇄2CO+2H_{2}\\ \Delta H_{298K}=+247.4\;kJ/mol \end{array}$$

5 $$\begin{array}{l} CH_{4}+\frac{1}{2}O_{2}⇄CO+2H_{2}\\ \Delta H_{298K}=-35.7kJ/mol \end{array}$$

6 $$\begin{array}{l} CH_{4}+H_{2}O⇄2CO+3H_{2}\\ \Delta H_{298K}=+206kJ/mol \end{array}$$

## 4 Effect of Biogas/Biomethane Contaminants on Catalytic Reforming: H_2_S and Nitrogen Species (NH_3_)

H_2_S can have a significant impact on CH_4_ reforming processes.^74^ H_2_S is a compound that is consequently generated in CH_4_ production, and its presence poses some problems during the use of CH_4_. The presence of H_2_S can poison the catalysts used in the process, such as Ni, reducing their catalytic activity and, consequently, the efficiency of CH_4_ and CO_2_ conversion.^75^ Additionally, H_2_S can react with the metals in the catalyst to form sulfides, changing the catalyst’s structure and ability to facilitate the reaction. This can lead to the permanent deactivation of the catalyst, requiring its replacement or regeneration, which increases operational costs.

Jablonski et al.^76^ discuss the mechanism of sulfur deposition on the catalyst surface. As mentioned earlier, H_2_S deposition can be related to the adsorption process of the contaminant on the metal surface. It can be observed that as the contaminant concentration increases, catalytic activity significantly decreases for both catalysts. To rigorously compare the deactivation of Ni/YSZ and commercial Ni/K_2_O–CaAl_2_O_4_ catalysts, the activity α, was used to relate the initial CH_4_ conversion to the deactivated CH_4_ conversion. For Ni/YSZ, in a reaction test at 750 °C, the catalytic activity decreased from 0.99 to 0.03 and 0.02 with 3 and 5 ppm of H_2_S, respectively. For Ni/K_2_O–CaAl_2_O_4_, the activity decreased from 0.82 to 0.24 and 0.17 with 3 and 5 ppm of H_2_S, respectively.

Table 1 presents a literature review on the impact of H_2_S addition on different CH_4_ reforming processes. This analysis includes the influence of H_2_S on SRM, ATR, and DRM, highlighting how this contaminant affects process efficiency, catalyst lifespan, and the integrity of pipelines and other system components.

**Table 1:** Summary of Techniques and the Effect of Sulfur Compounds on Catalytic Reforming

Catalytic reforming	H_2_S concentration (ppm)	Sulfur compounds effect	Ref
Dry reforming	10 ppm	Experiments were conducted at 700 and 800 °C with 5 and 10 ppm of H_2_S in a synthetic biogas with CH_4_/CO_2_ ratios of 1.5 and 2.0. The poisoning and deactivation of the catalyst increased with the H_2_S concentration due to sulfur adsorption on the metal surface. The reduction in efficiency was about 20%.	^74^
	2 and 50 ppm	Tests of the catalysts were conducted in a tubular reactor at 750 °C and 1 atm, both in the absence and presence of 2 and 50 ppm of H_2_S. The findings suggested that at 750 °C, H_2_S could potentially undergo decomposition into H_2_ and elemental sulfur across all catalysts. Deactivation of the bimetallic catalyst primarily resulted from sulfate species formation.	^77^
	3.2 ppm	H_2_S tests were conducted at 800 °C and the molar ratio of CH_4_:CO_2_:H_2_S = 1:1:0.17 using Ni/A–H catalyst. H_2_S leads to catalyst deactivation, due to the chemisorption of sulfur compounds on the Ni sites. As a result, CH_4_ conversion was reduced by around 46% by H_2_S poisoning. With the interruption of H_2_S supply, it is possible to have a slight reactivation of the catalyst (around 12% of the activity can return).	^78^
	20 ppm	Ni/MgAl, NiCo/MgAl, Ni/MgAl@CeO_2_ and NiCo/MgAl@CeO_2_ catalysts were tested to evaluate the effect of 20 ppm of H_2_S on synthetic biogas with molar ratio of CO_2_:CH_4_ = 1:1. Without H_2_S all the catalysts showed around 93% of CH_4_ conversion and with the exposure the activity instantaneously reduced to around 80%, and after a few minutes of reaction to 18%, as a result of catalyst poisoning and metal sintering over time. After stopping supplying H_2_S, CH_4_ reforming can increase a little again (from 18 to 23%) as a result ofdesorption/hydrogenation of sulfur.	^79^
Steam reforming	1, 3, and 5 ppm	The authors evaluated the insertion of 1, 3, and 5 ppm of H_2_S in the DRM and SRM processes and found that H_2_S could accelerate the SRM process deactivation. For Ni/YSZ catalyst the activity decreased from 0.99 to 0.03 and 0.02. For Ni/K_2_O–CaAl_2_O_4_, the activity decreased from 0.82 to 0.24 and 0.17.	^76^
	150, 350, 500, and 1000 ppm	Effect of H_2_S on biogas sorption-enhanced SRM using a Pd/Ni–Co catalyst and dolomite as a CO_2_ sorbent. The H_2_S concentrations used were 150, 350, 500, and 1000 ppm. The concentrations of 500 and 1000 ppm decreased the reaction time by approximately 10.8% and 4.5%, respectively, over a 40 min period.	^80^
	0–200 ppm	The impact of sulfur poisoning was evaluated over Ni/Al_2_O_3_, Rh/Al_2_O_3_ and Ni/Mayenite catalysts. It was observed that 10 ppm of H_2_S leads to complete catalyst deactivation and reduced conversion of CH_4_.	^81^
	50 and 500 ppm	Ni/LaSrMnO_4_ was evaluated in relation to H_2_S poisoning during SRM. It was found that with 50 ppm of H_2_S the catalyst was irreversibly poisoned in tests done at 850 °C and 8 h of reaction. The effect of H_2_S was described by two mechanisms: (i) when H_2_S molecules come into contact with the Ni surface, there is the dissociative adsorption of H_2_S. The resulting sulfur radicals are strongly adsorbed, forming a monolayer that rapidly covers and blocks the metallic active sites. This subsequently inhibits the adsorption, dissociation, oxidation, and diffusion of CH_4_ and H_2_O necessary for the SRM; (ii) gradual degradation of the material and the formation of inactive Ni/S compounds, primarily Ni_3_S_2_.	^82^
Autothermal reforming	50 ppm	For the catalytic activity test in the presence of sulfur, 50 ppm of H_2_S was added to the gas. The authors identified a higher resistance of perovskite catalysts to sulfur poisoning. The studies show a decrease of only 20% in H_2_ yield.	^83^
	50, 100, and 200 ppm	Syngas containing CH_4_:H_2_O:CO_2_:O_2_= 1:0.66:0.33:0.2 was used to evaluate the effect of H_2_S in the ATR, using NiO and CaAl_12_O_19_ catalysts. Catalysts activity decreased to 45.1% after 7h of measurement with sulfur compounds. From both conditions, the activation occurs fast, in the first reaction minutes, since H_2_S is preferentially adsorbed at the most active Ni sites of the catalyst, causing a significant decrease in catalyst activity, particularly at the onset.	^84^

Recent studies highlight the significant impact of H_2_S on catalytic processes during biogas reforming. Pawar et al.^74^ demonstrated that H_2_S concentrations of 5 and 10 ppm during dry reforming with a Ni/Al_2_O_3_ catalyst reduced gas conversion efficiency by approximately 20% and led to coke formation. Their work showed that removing H_2_S increased gas conversion by 25% to 35%. Similarly, Akansu et al.^77^ investigated manganese incorporation in Ni-based catalysts for dry reforming of biogas, demonstrating that manganese promoted CO_2_ conversion while offering no significant improvement in sulfur resistance, as indicated by sulfur component formation like COS (carbonyl sulfide) and H_2_O. Philipp Wachter et al.^84^ explored the effects of H_2_S and SO_2_ on a commercial Ni-based catalyst, reporting that both contaminants rapidly led to catalyst deactivation. A pronounced decrease in catalytic activity was observed across all tested H_2_S concentrations, with the most significant decline occurring at 200 ppm. Under this condition, activity dropped markedly from 100% to 45.1% after just 7 h of operation.

Biogas and biomethane, after biological desulfurization, contain minor components like H_2_S, which, even in low concentrations, can interact with the metallic phase of catalysts.^85,86^ This interaction can lead to catalyst inactivation, affecting the longevity of reformer reactor tubes and resulting in additional costs for replacing the catalyst bed. The complete elimination of H_2_S for reforming systems is extremely important, as even very low concentrations of this contaminant are capable of poisoning catalysts, drastically reducing their activity and lifespan.^78,87^ Therefore, the implementation of efficient sulfur removal technologies becomes essential, especially to ensure operational stability and durability of the reforming CH_4_ processes previously discussed. Furthermore, the development and use of highly efficient and H_2_S-tolerant catalysts are crucial to mitigate the negative impacts of this contaminant, ensuring performance and industrial feasibility in the conversion of CH_4_ into syngas or other products of interest.

Current understanding of N_2_ influence on dry, steam, and autothermal reforming remains limited. N_2_ can be present in the biogas/biomethane composition due to the biological desulfurization with air injection. In catalytic reforming, N_2_ can react with feed gas species or directly with catalyst surfaces. Nevertheless, in both cases, the influence is due to the formation of NH_3_ from N_2_. N_2_ is inert and, therefore, will directly affect the reaction process only under particular conditions; even so, its presence can also modify the partial pressures of the other gaseous species in the equilibrium system. The presence of N_2_ in biogas can hinder grid injection, pipelines, equipment performance, and storage systems. Additionally, N_2_ reduces the yield of reforming products by occupying part of the gas composition without contributing to the reaction.^80^ Table 2 provides some information about the effect of N_2_ on steam, dry, and autothermal reforming.

**Table 2:** Summary of Techniques and the Effect of N_2_ Compounds on Catalytic Reforming

Catalytic reforming	N_2_ concentration (%)	Effect	Ref
Dry reforming	20	For a synthetic biogas composition of proportions CH_4_:CO_2_:N_2_ = 2:2:1, under specific operating conditions with Ni catalysts on different supports, the conversion rate (for CH_4_) varied from 13% to 95% (H_2_/CO between 0.9).	^88^
	9	The test was conducted using a 5/5/1 (CH_4_/CO_2_/N_2_) ratio with a Ni catalyst supported on a mix of α-Al_2_O_3_ and TiO_2_–P25. N_2_ played a crucial role in the experiment, serving both as a cooling agent for the reactor and as a dilution gas during the reaction. It controlled the temperature and the concentration of reactants, ensuring the stability and reproducibility of the results, with a carbon balance accurate within ±4%.	^89^
Steam reforming	86–91	The study investigated steam CH_4_ reforming using a 20Ni/SiC–M monolithic catalyst at 600 °C. The process was conducted in a fixed-bed reactor, with gases premixed and injected in pulses. N_2_ was used as a carrier gas and for gas dilution. The authors did not identify a significant influence of N_2_, as it remained inert during the process.	^90^
	7.0	For reactions using Ru, Pt, Rh and Ir catalysts, with a feed of N_2_/CH_4_/H_2_O = 1/4/10, up to 81 ppm of NH_3_ was obtained in the final gaseous product, corroborating the reduction in H_2_ produced.	^91^
	0.4–70	Authors evaluated the effect of 0.4% to 70% of N_2_ on steam CH_4_ reforming of a gas with 80% to 27% of CH_4_, with commercial catalyst (NiO on Al_2_O_3_ support). While the presence of inert N_2_ reduces the partial pressure of the reactants, which is advantageous in steam reforming, a high concentration of inert gases increases the energy costs of operating the reforming plants. As N_2_ increases in the feed, the enhancement on H_2_ yield decreases.	^92^
Autothermal reforming	20	The presence of N_2_ in the process can hinder the reforming process and lead to its adherence in the composition (CH_4_:CO_2_:N_2_ = 2:2:1) of the synthesis gas produced by the reaction, which can result in a product gas with a larger volume, generating more significant problems with compression and storage.	^93^

As shown in Table 2, there are various negligible or non-negligible impacts that biogas/biomethane containing N_2_ can have on catalytic reforming. According to Watanabe et al.,^91^ even though N_2_ is known to be stable since it is inert, there is a risk of NH_3_ production from the N_2_ impurity present in biomethane and/or natural gas in conditions with temperatures above 500 °C. NH_3_ leads to catalyst degradation and is formed by the reaction of N_2_ with water (H_2_O) from the catalytic steam reforming (eq 7). NH_3_ can also be produced by the reaction between N_2_ and CH_4_ (eq 8). Unlike N_2_, NH_3_ is not an inert gas and contributes to the catalyst degradation and coke deposition (eq 8). Thus, in the case of using the syngas in a FC, NH_3_ also affects the electrode and the whole system.

7 $$5N_{2}+6H_{2}O\to 4NH_{3}+6NO,\left(\mathrm{balanced equation}\right)$$

8 $$2N_{2}+3CH_{4}\to 4NH_{3}+3C,\left(\mathrm{balanced equation}\right)$$

Moreover, N_2_ can lead to a lower synthesis conversion yield in cases where it does not react with H_2_O and/or CH_4_. In this case, the syngas obtained at the end of the process has less H_2_ since N_2_ took its place in the composition of the final gas obtained.^93^ Brus et al.^94^ also noted higher N_2_ and lower H_2_ in the obtained syngas when using CH_4_ blended with N_2_. Thus, the presence of N_2_ in the synthetic reagent mixture directly affects the partial pressure of the components. In this case, different stoichiometries for the feed gas were evaluated: CH_4_/N_2_/H_2_O of 1:3:3; 1:6:2.5; 1:4:5; 1:2:4; 1:1:3, resulting in a decrease in CH_4_ conversion rates directly proportional to the increase in N_2_ concentration.

Consequently, the presence of N_2_ in the feed gas mixture can lower the synthesis conversion yield by displacing H_2_ in the final syngas composition, resulting in less H_2_.^93^ Studies indicate that higher N_2_ concentrations lead to lower H_2_ levels and decreased CH_4_ conversion rates, proportional to the N_2_ concentration.^94^ In ATR, N_2_ remains inert, increasing the gas volume and complicating storage, a phenomenon also observed in DRM and SRM, where biogas with high N_2_ content produces syngas with up to 20% N_2_.^95^ However, N_2_ can also enhance the reforming process. Chein et al.^96^ found that it slightly increases CH_4_ and CO_2_ conversion rates at low pressures by shifting the equilibrium toward decreasing pressure, thus promoting the reaction. The accumulation of nonreactive N_2_ in syngas can have both positive and negative effects on FCs, which, along with the impact of H_2_S, will be discussed in the next section.

## 5 High Efficiency Energy Generation in FCs

FCs are efficient electrochemical devices for renewable energy generation, increasingly applied in systems using biogas, biomethane, or renewable H_2_.^97,98^ Contaminants are deposited at the edge of FCs, at the interface between the electrode and the electrolyte, where the chemical reactions that generate electrical voltage occur. Contaminants tend to accumulate at the electrode/electrolyte interface (TPBs: triple phase boundaries) in FCs, precisely where the electrochemical reactions responsible for voltage generation take place, reducing cell efficiency and leading to catalyst failure.^99,100^ SOFCs and PEMFCs are generally considered promising candidates for widespread future applications. However, unlike PEMFCs, which employ proton exchange membranes as electrolytes, SOFC utilizes solid oxides, making them all-solid-state FCs. The proton exchange membrane in PEMFC is prone to corrosion and failure. Whereas SOFC typically exhibits a longer lifespan. Moreover, PEM requires precious metal catalysts such as Pt during operation, while SOFC does not, reducing operating costs.^101^ Additionally, SOFC operates at medium to high temperatures (600 to 1000 °C), compared to the lower operating temperatures of PEMFCs (60 to 100 °C). This higher temperature range enhances the electrochemical reaction rate, increasing energy conversion efficiencies. This advantage can be further amplified by implementing a cogeneration system that utilizes the abundant thermal energy in the SOFC exhaust gas. SOFC also has better power density, versatile types of fuels can be used, hydrocarbons can be directly consumed, and due to the high operating temperatures, residual heat from the process can be recycled to increase the system energy efficiency by up to 90%. Therefore, given its high-power density, SOFC presents significant advantages over the other FCs in applications that require large-scale power generation equipment. These applications include distributed power generation.^102^

Syngas produced through catalytic reforming can be used as fuel in SOFCs for power generation without the necessity of upgrading to obtain H_2_. In addition to reducing equipment and operating costs, the coupling of biogas and biomethane catalytic reforming with an SOFC leads to an efficient waste-based CHP system, increasing the energy efficiency of electricity production in a biogas plant. The general gaseous syngas impurities are carbonaceous, sulfurous, and nitrogenous species, like CO_2_, H_2_S, and N_2_, respectively. Even though SOFCs have tolerance to contaminants, these substances can lead to carbon deposition on the surface anode of the FCs. Besides, higher concentrations of unreacted portions of CH_4_, CO_2_, and CO lead to greater deposition since the interaction between carbon and the FC catalyst induces a catalytic CH_4_ cracking reaction that leads to different coking distributions in the anode area. In addition to carbon formation, sulfur and nitrogenous compounds are another obstacle for a stable, long-term, and high-performance SOFC operation.^103,104^ The next topic will discuss the effects of H_2_S and N_2_ on SOFCs.

## 6 Effects of Sulfur and Nitrogenous Compounds on SOFC Performance

### 6.1 Sulfur Compounds

Sulfur compounds are naturally present in biogas. Their concentration varies widely depending on the biogas source and the desulfurization processes employed. As has been demonstrated, prior to utilizing biogas in an integrated fuel cell (FC) electricity generation system, desulfurization is applied with the objective of reducing H_2_S concentrations. Thus, the concentration of H_2_S in biogas, biomethane, and syngas commonly ranges from 100–5000 ppm, <7 ppm, and <1 ppm, respectively. It means, even with biogas pretreatment, the H_2_S remains even in low concentrations. Several studies have found that H_2_S is poisonous and degrades SOFC performance even at a few ppm between 50 ppb up to 1 ppm. At moderate levels, the poisonous effect of H_2_S can be reversible. However, at high concentrations, H_2_S causes irreversible damage to the SOFC by anode poisoning.^63^ In addition, other contaminants present in biogas, such as N_2_, can act as an inert for catalytic steam reforming and SOFC. However, some catalysts can reduce N_2_ with H_2_ to NH_3_ in the gas phase, which can induce negative effects in FCs. When N_2_ is not reduced, it can have positive effects as a fuel carrier in FCs.^105^ The effect of sulfur and nitrogenous compounds will be discussed below.

Almost all sulfur species in gases such as syngas, natural gas, biogas, and/or biomethane are eventually converted into stable H_2_S, a significant environmental pollutant. Even at low concentrations (2 ppm), sulfur compounds may promote a significant poisoning effect on fuel cell anodes, which is a crucial obstacle in the use of SOFCs. The effects of sulfur compounds in anodes occur in two steps: first, a fast degradation over several seconds happens due to the dissociative H_2_S adsorption on the anode catalyst surface, commonly Ni. Second, a gradual, slow degradation occurs over a prolonged period due to the accumulation of H_2_S on the anode catalyst surfaces.^106^

The scheme in Figure 4a represents the process occurring between the anode and the electrolyte during SOFC operation.^107^ In general, it can be divided into four steps: (1) H_2_ adsorption, (2) H_2_ dissociation and surface diffusion, (3) charge transfer accompanied by H_2_O formation, and finally, (4) H_2_O desorption. However, when H_2_S is present in the system, it preferentially adsorbs onto the Ni surface, inhibiting steps (1) H_2_ adsorption and (2) dissociation and surface diffusion, as shown in Figure 4b. Additionally, due to the relatively large size of H_2_O molecules, it is reasonable to expect that adsorbed sulfur near the TPB would also significantly slow down step (3) charge transfer, as this process requires available active sites for H_2_O generation.

**Figure 4:**
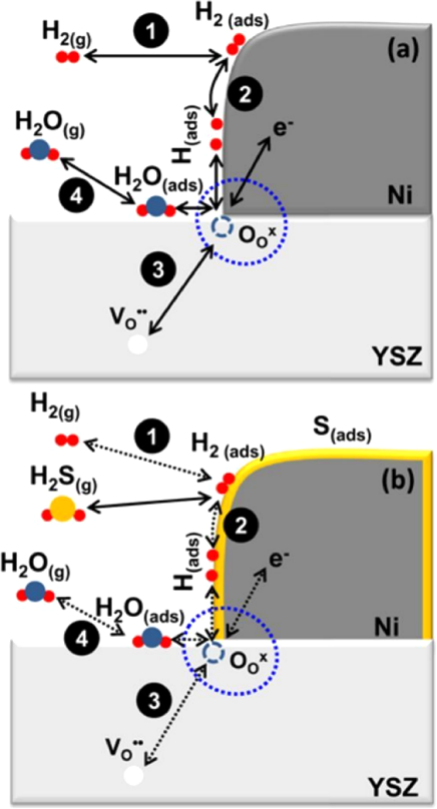
Schematic of Ni anode reactions for a conventional SOFC in the absence (a), and presence (b) of H_2_S. Reproduced with permission from.^107^ Copyright 2018 IOP Publishing.

The chemical reactions occurring during H_2_S operation in a Ni-based anode SOFC (Figure 4b) can be primarily described by two distinct processes: chemisorption and sulfidation.^108^ Reactions (eqs 9–11) illustrate how gaseous H_2_S dissociates into sulfur and H_2_, followed by its adsorption onto the Ni catalyst, forming Ni sulfide (Ni_*x*_S_*y*_) and leading to catalyst poisoning.^109–111^

9 $$H_{2}S_{\left(g\right)}⇄HS_{\left(ads\right)}+3H_{\left(\mathrm{ads}\right)}⇄S_{\left(\mathrm{ads}\right)}+H_{2\left(\mathrm{ads}\right)},\left(\mathrm{chemisorption}\right)$$

10 $$Ni+H_{2}S⇄NiS+H_{2},\left(\mathrm{sulfidation}\right)$$

11 $$3Ni+xH_{2}S⇄Ni_{3}S_{x}+xH_{2},\left(\mathrm{sulfidation}\right)$$

Sulfurs effect on FCs also depends on temperature since it hinders the electrochemical oxidation of H_2_ and polarization. The significant impact of sulfur on the catalytic activity of metal at low sulfur concentrations is likely due to the high isosteric heat of adsorption for H_2_S on the metal.^104^ This means that the low sticking effect of H_2_S on metal allows H_2_S to quickly reach the anode/electrolyte interface.^112^ There, it adsorbs on the metal of the anode, blocking the adsorption of other active species and thus inhibiting the electrochemical oxidation of H_2_.^111,113^ Vivet et al.,^114^ described this electrochemical sulfur poisoning (eqs 12–13) as

12 $$H_{2}S_{\left(g\right)}+O^{2-}\to H_{2}O_{\left(g\right)}+S_{\left(on\;Ni-TPB\right)}+2{e^{-}}_{\left(on\;Ni\right)},\left(\mathrm{adsorption}\right)$$

13 $$H_{2}S_{\left(g\right)}\to H_{2\left(g\right)}+S_{\left(on\;Ni\right)},\left(\mathrm{adsorption}\right)$$

As previously discussed, conventional Ni/YSZ anodes are highly susceptible to sulfur-induced deactivation, even at trace concentrations, leading to substantial performance degradation.^115^ This deactivation arises from the strong adsorption of sulfur species on the nickel (Ni) surface, which blocks the active sites required for the electrochemical oxidation of the fuel and increases the anodic polarization. To elucidate this phenomenon, Kim et al.^116^ conducted comprehensive investigations using surface-enhanced Raman spectroscopy (SERS), demonstrating that the accumulation of S–S bonds at the sulfur-contaminated solid–gas interface severely impairs the CH_4_ reforming process. Figure 5a presents a schematic representation of the sulfur poisoning mechanism in Ni/YSZ anodes, while Figure 5b shows in situ SERS data with temporal resolution for an anode exposed to 100 ppm of H_2_S at 500 °C. A pronounced peak at ∼470 cm^–1^, assigned to S–S bonds, was observed to increase in intensity with prolonged exposure, thereby confirming the progressive sulfur poisoning of the anode. Figure 5c illustrates the Ni sulfidation mechanism that occurs after cooling of the Ni/YSZ anode, which was further validated by ex-situ Raman analysis of the exposed electrode (Figure 5d). Electrochemical analyses (Figure 5e–g) further confirmed the H_2_S poisoning in CH_4_ and its detrimental impact on both the total cell resistance and power density. To further substantiate this conclusion, Mai Thi et al.^117^ successfully demonstrated the temporal evolution of Ni_3_S_2_ over approximately 47 h using in situ Raman spectroscopy (Figure 5h). In their experiments, 200 ppm of H_2_S was introduced during the operation of a SOFC equipped with a Ni/YSZ anode, operated in open-circuit voltage (OCV) mode at 500 °C, under a 3% H_2_/3% H_2_O/Ar atmosphere on the anode side. The characteristic Ni_3_S_2_ band pairs at ∼190–200 cm^–1^ and ∼290–340 cm^–1^ became discernible after approximately 7.4 h of operation, progressively increasing in intensity until ∼17.3 h, after which the signal reached a plateau. This saturation phenomenon is consistent with the optical observations reported by Cheng et al.^118^ (Figure 5i–j), which reveal that the Ni_3_S_2_ phase (black regions) progressively consumes the metallic Ni phase (gray regions).

**Figure 5:**
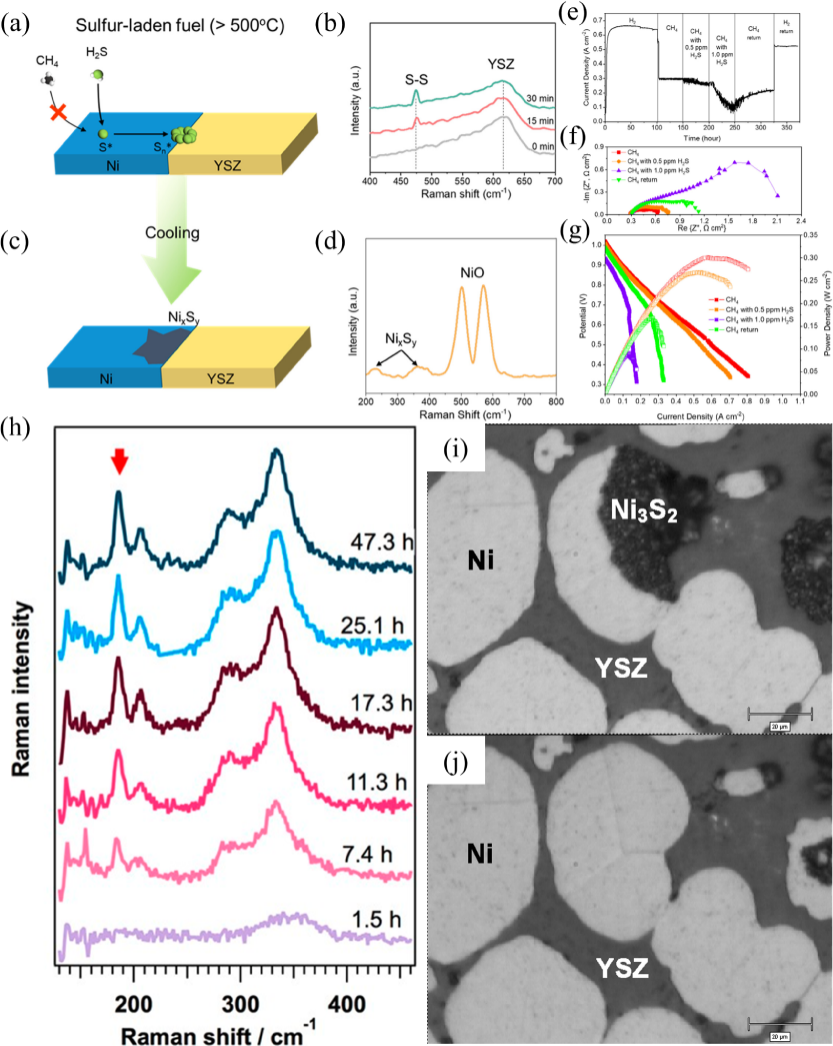
(a) Schematic representation of the sulfur poisoning mechanism in Ni/YSZ anodes. (b) *In-situ* SERS analysis of the anode exposed to 100 ppm of H_2_S. (c) Schematic representation of the Ni sulfidation in cooled Ni/YSZ anodes. (d) Ex-situ Raman spectrum of Ni sulfidation in cooled Ni/YSZ anodes. (e) Long-term electrochemical test of a SOFC with different fuels showing impact of H_2_S poisoning on the anode. (f) Impedance spectra of the SOFC at 700 °C with CH_4_ as fuel and the impact of H_2_S presence on the total resistance. (g) *I*–*V* curves of the SOFC at 700 °C with CH_4_ as fuel and the impact of H_2_S presence on the OCV potential and power density. Reproduced with permission from^116^ Copyright 2021 American Chemical Society. (h) *In-situ* Raman spectra of the anode surface in different exposure times to 200 ppm of H_2_S at 500 °C in OCV mode. Reproduced with permission from Mai Thi et al.^117^ Copyright 2017 WILEY-VCH Verlag GmbH & Co. KGaA, Weinheim. Optical microscopy images of the Ni/YSZ anode (i) with sulfur contamination, and (j) without sulfur contamination. Reproduced with permission from Cheng et al.^118^ Copyright 2007 American Chemical Society.

In this sense, the search for new sulfur-tolerant materials has become critically important for advancing biogas-operated SOFCs.^119,120^ Table 3 presents the selected comparison of sulfur tolerances based on different anodes.

**Table 3:** Comparison of Sulfur Tolerances in Different SOFCs-Anode Types^a^,^b^,^c^,^d^,^e^,^f^,^g^

H_2_S concentration (ppm)	*T* (°C)	Anode	Observation about the performance	Ref
0.05, 0.5, and 2	750, 900 and 1000	Ni/YSZ cermet	Reduced current density and cell voltage, leading to a loss of efficiency due to poisoning.	^121^
2–100	850	Ni/YSZ cermet	The effect of H_2_S was directly on the surface of the anode, with a process of Ni adsorption reducing the efficiency of the cell.	^108^
5	800	LDC/LST	The contaminated syngas decreases the area specific resistance (ASR_pol_) of the anode from 2.1 Ω·cm^2^ to 1.8 Ω·cm^2^.	^122^
20	700, 800 and 900	Ni	The interaction of sulfur and Ni was favored, forming Ni sulfide (Ni_3_S_2_) even at low H_2_S concentrations.	^123^
0–300	900	SYT and SYTN	For the SYT anode, the maximum power densities decreased slightly by 7% (32.4 mW/cm^2^ at 0 ppm to 30.1 mW/cm^2^ at 300 ppm). For the SYTN anode, the impact of poisoning was higher, with power reductions of 46% (42.9 mW/cm^2^ at 0 ppm and 23.1 mW/cm^2^ at 300 ppm).	^124^
1	900	Ni/GDC	The performance of SOFCs during steam CH_4_ reforming was investigated in the presence of H_2_S. They observed that both catalytic performance for steam CH_4_ reforming and the electrochemical properties for H_2_ electro-oxidation of a Ni/GDC anode decreased drastically in the presence of 1 ppm of H_2_S.	^125^
∼2–24	850	Ni/YSZ and Ni/ScYSZ	The gas mixture used in the tests contained 13% of H_2_, 29% of CH_4_, and 58% of H_2_O. H_2_S concentrations were tested between 2 and 24 ppm. The SOFC anodes were degraded by up to 40%.	^126^
5	800	NiO/ScSZ	The effects of sulfur poisoning were studied in an experiment that covered a wide range of operating conditions. The study of the initial voltage changes across different current densities (0.2, 0.4, 0.6, and 1.0 A/cm^2^) using fuels with 5 ppm of H_2_S revealed that sulfurs impact on anode performance intensifies as current density increases.	^127^
0.125	800	Ni/ScSZ	The assessment of H_2_S impact on internal dry reforming in biogas-fueled SOFCs demonstrated that even at a concentration as low as 0.125 ppm, H_2_S severely impairs CH_4_ reforming efficiency in FC. At 800 °C, the reforming efficiency decreased from over 95% to below 10% within 10 h by the H_2_S poisoning effect.	^128^

a **Ni/YSZ:** Ni–yttria stabilized zirconia.

b **LCO/LST:** La_0.4_Ce_0.6_O_1.8_–La_0.4_Sr_0.6_TiO_3 ± δ_.

c **SYT:** Sr_0.92_Y_0.08_TiO_3−δ_.

d **SYTN:** Sr_0.92_Y_0.08_Ti_1–x_Ni_x_O_3−δ_.

e **Ni/GDC:** Ni/gadolinia-doped ceria.

f **NiO/ScYSZ:** Nickel oxide/yttrium doped-scandium stabilized zirconia.

g **Ni/ScYSZ:** Ni–Yttrium-doped scandium stabilized zirconia.

Table 3 summarizes some of the effects of H_2_S concentrations on the performance of SOFC anodes under different conditions. In general, key findings from these studies highlight the significant impact of H_2_S on cell efficiency, catalytic activity, and overall anode performance. It also demonstrates that even low concentrations of H_2_S can significantly degrade the performance of SOFC anodes. The level of impact varies depending on the type of anode material, H_2_S concentration, and operating temperature. Ni-based anodes (Ni, Ni/YSZ, Ni/ScYSZ, Ni/ScSZ) are particularly susceptible to sulfur poisoning, which leads to the formation of Ni sulfide and substantial efficiency losses. The sensitivity of different anode materials to H_2_S also varies, with SYTN anodes showing greater susceptibility compared to SYT anodes.

In this context, knowing that Ni-based anodes are highly susceptible to degradation in the presence of sulfur, new materials have been developed to mitigate this degradation.^129^ For instance, La_0.8_Sr_0.2_Fe_0.9_Nb_0.1_O_3−δ_ perovskite-based anodes exhibit increased resistance to H_2_S and possessed reversible properties after exposure. Figure 6a shows the performance stability of the LSFNb/SDC/ScSZ/LSM/ScSZ cell at 800 °C under a constant current density of 0.4 A/cm^2^ while being exposed to 200 ppm of H_2_S. Initially, there is a slight performance promotion, likely due to the formation of functional FeS nanoparticles that enhance catalytic activity. However, this is followed by a rapid degradation phase lasting approximately 30 min, where the cell voltage drops from 0.835 to 0.732 V. This behavior indicates that the anode is significantly affected by sulfur poisoning, leading to a decrease in electrochemical performance. In Figure 6b, the time dependence of the *I–V* polarization curves show a clear trend of performance degradation over time. The maximum power densities (MPDs) decrease from 0.44 W/cm^2^ to 0.29 W/cm^2^ after 50 h of exposure to H_2_S. This decline in MPD correlates with the observed voltage drop, indicating that the anodes ability to facilitate electrochemical reactions is compromised due to the accumulation of sulfur species on its surface. Figure 6c presents the EIS spectra of the LSFNb anode at different time points during the sulfur poisoning process. The Nyquist plot reveals that the spectra in high-frequency resistance increase over time, indicating a deterioration in the charge transfer process. Specifically, EIS spectra in high-frequency resistance rises from 0.064 Ω·cm^2^ at the beginning to 0.126 Ω·cm^2^ after 50 h of exposure to H_2_S. Conversely, the EIS spectra in low-frequency resistance shows a decreasing trend, suggesting that the gas adsorption and dissociation capabilities of the anode are somewhat maintained despite the sulfur poisoning. This behavior indicates that while the anode is affected by sulfur, it retains some level of activation. Figure 6d provides further insights into the recovery of the LSFNb anode after the interruption of H_2_S exposure. The EIS spectra before and after turning off the H_2_S flow shows a notable decrease in both high and low-frequency resistance, indicating that the charge transfer and gas adsorption processes can recover when the sulfur source is removed. This recovery suggests that the sulfur species formed on the anode surface are not permanently damaging and can be alleviated by stopping the exposure to H_2_S, showing sulfur poisoning in the LSFNb anode. The initial performance promotion, followed by rapid degradation, highlights the dual role of sulfur species, where they can initially enhance performance through the formation of FeS nanoparticles but ultimately lead to significant performance loss due to the coverage of active sites. The EIS results further emphasize the anode ability to recover from sulfur poisoning, showcasing its potential for sulfur tolerance in practical applications. This understanding of sulfur poisoning mechanisms is crucial for the development of more resilient SOFCs.

**Figure 6:**
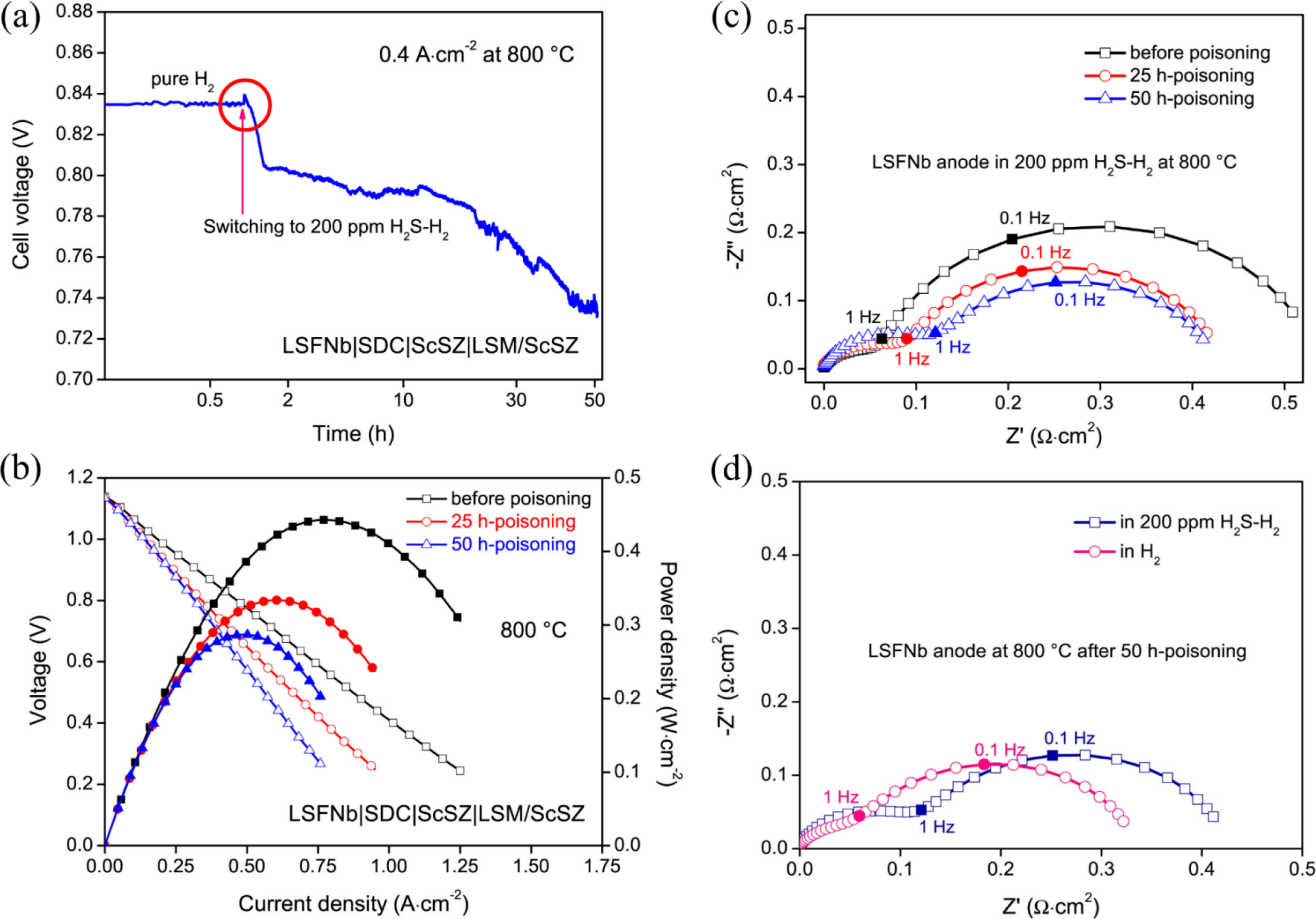
(a) SOFC performance stability at 800 °C fueled by H_2_S/H_2_. (b) *I–V* curves of the whole cell. (c) EIS spectra of LSFNb anode at 25h and 50h of the sulfur poisoning at 800 °C fueled by H_2_S/H_2_. (d) EIS spectra of LSFNb anode before and after turning off the H_2_S after being poisoned for 50 h. Reproduced with permission from Li et al.^129^ Copyright 2020 Elsevier B.V. All rights reserved.

In another important study, Price et al.^130^ developed LSCT anodes impregnated with high-cost composite catalysts such as Pt/CGO and Rh/CGO, which proved to be highly promising in the presence of H_2_S. Additionally, the study showed that the Ni/CGO catalyst experienced severe sulfur poisoning, while the Pt- and Rh-based catalysts remained stable. The study investigated H_2_S concentrations ranging from 1 to 8 ppm in humidified H_2_. The Ni catalyst was severely affected by just 1.0 ppm of H_2_S, while the Rh catalyst remained stable in the presence of 2 ppm of H_2_S. The Pt-based catalyst demonstrated the best stability, remaining unaffected by 8 ppm of H_2_S. Both noble catalysts, Pt/CGO and Rh/CGO, exhibited excellent recovery after the H_2_S supply was interrupted. In another study on Rh-ceria composite catalysts, stability was reported with 50 ppm of H_2_S.^133^ This stability was attributed to the sulfidation of ceria, which likely served as a “sacrificial” site for sulfur adsorption and reaction.^131^ This process enabled the Rh catalytic component to maintain its catalytic activity after the removal of H_2_S.

In addition to the development of novel anode materials, alternative strategies such as the introduction of H_2_O into the fuel stream for the removal of sulfur species have also been investigated.^107,115,116,132–135^ The addition of H_2_O, typically in the form of steam, has emerged as a promising approach to mitigate sulfur poisoning in SOFCs, operating through different mechanisms and achieving varying degrees of effectiveness depending on the specific anode material employed.^132^ For conventional Ni/YSZ anodes, the presence of steam in the fuel can slow down the sulfur poisoning process and mitigate performance degradation, particularly under high H_2_S concentrations. Beyond SOFC conditions, catalytic studies have shown that H_2_O promotes the desorption of SO_2_.^115^ Moreover, the regeneration of Ni-based catalysts poisoned by sulfur can be accomplished through thermal treatments in steam atmospheres at temperatures between 800 and 900 °C, enabling the removal of a significant fraction of the sulfur as H_2_S.^115^ Nevertheless, sulfur poisoning of Ni/YSZ anodes remains a persistent challenge, even in the presence of H_2_O.

A more effective approach to H_2_O-mediated sulfur tolerance involves replacing the YSZ phase in cermet anodes with mixed ionic–electronic oxides that also exhibit proton conductivity, such as Ba­(Zr,Ce)­O_3_-based materials.^107^ Compositions such as BaZr_0.1_Ce_0.7_Y0.2–_*x*_Yb_*x*_O_3_ (BZCYYb) display a strong propensity for H_2_O adsorption and simultaneously conduct both protons and oxide ions. The dissociative adsorption of H_2_O on the surfaces of these materials is considerably more favorable than on ZrO_2_- or CeO_2_-based oxides. In this context, Yang et al.^134^ reported a high-performance BaZrCeYYb mixed ionic conductor that exhibited remarkable resistance to sulfur deactivation. They further demonstrated that this enhanced sulfur tolerance may be associated with the catalytic activity of the anode component, which promotes both sulfur oxidation and the adsorption of H_2_O introduced into the fuel stream. Long-term durability tests of 200 and 1000 h at 750 °C, under exposure to 30 and 10 ppm of H_2_S in H_2_/3%H_2_O, respectively, revealed negligible degradation of the electrochemical potential.

A more comprehensive analysis by Kim et al.^135^ demonstrated that the H_2_O-mediated sulfur tolerance mechanism of a proton-conducting oxide (Figure 7a) represents a promising pathway to enhance sulfur resistance in SOFCs. As shown in Figure 7b, when dry H_2_ containing 100 ppm of H_2_S was introduced to a Ni/BZCYYb anode at 500 °C, *a* −SO_4_ band at ∼980 cm^–1^ rapidly emerged in the *in situ* SERS spectra of the BZCYYb surface, indicating sulfur adsorption and oxidation. In the absence of H_2_O vapor, the −SO_4_ species remained on the surface, as reflected by the integrated peak intensity over time. However, upon replacing dry H_2_ with humidified H_2_ containing 3% H_2_O, the −SO_4_ band at ∼980 cm^–1^ decreased markedly. This observation suggests that H_2_O facilitates the removal of SO_4_ species from the BZCYYb surface, indicating that H_2_O adsorption may serve as an effective mechanism for eliminating sulfur species from SOFC anodes. EIS measurements (Figure 7c), carried out under both dry and humid conditions, were consistent with the Raman intensity data (Figure 7d). The electrode polarization resistance was significantly higher in dry H_2_ with 100 ppm of H_2_S where greater −SO_4_ formation was observed, and decreased substantially in the presence of H_2_O vapor, coinciding with the removal of −SO_4_. Collectively, these findings support the hypothesis that −SO_4_ is the primary functional group responsible for increased electrode polarization under sulfur-containing atmospheres. In summary, the results demonstrate that H_2_O plays a critical role in mitigating sulfur poisoning in SOFC anodes based on proton-conducting oxides such as BZCYYb.

**Figure 7:**
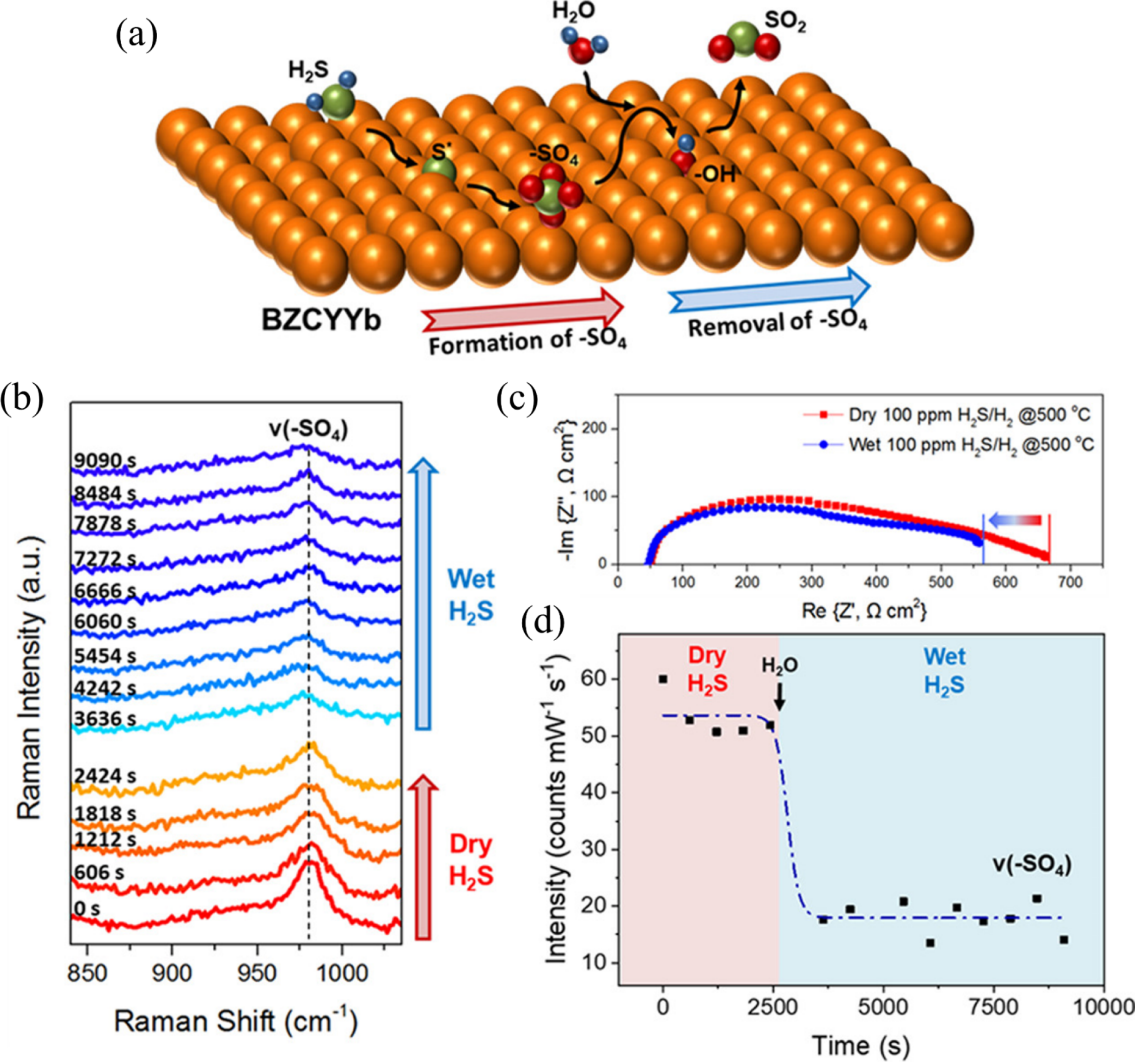
(a) Schematic representation of the H_2_O-mediated formation and removal of $SO_{4}^{-}$ at a proton-conducting oxide interface. (b) *In-situ* SERS analysis of a Ni/BZCYYb anode exposed to 100 ppm of H_2_S under dry and humid atmospheres. (c) Electrochemical impedance spectra of the Ni/BZCYYb anode operating with H_2_ containing 100 ppm of H_2_S at 500 °C under dry and humid conditions. (d) Peak intensities obtained from the *in-situ* SERS analysis in (b), corresponding to $SO_{4}^{-}$ species under dry and humid atmospheres. Reproduced with permission from.^135^ Copyright 2019 American Chemical Society.

In summary, when a SOFC anode is operated with H_2_S-containing syngas, the impurities may promote: (i) the reduced mass transport of the fuel gas molecules, since it is hindered due to the adsorption of impurities on the anode surface, which clogs gas diffusion channels; (ii) the catalytic ability of the anode toward chemical and electrochemical reactions is diminished as impurity atoms on the surface poison and deactivate the TPBs of the FC; (iii) the ability of the anode to transport O^2–^ is compromised by the formation of other phases; (iv) the electrical conductivity of the anode is also affected. Furthermore, the conductivity at the interconnect-anode interface is impacted, and the structural integrity of the anode and sealing materials is compromised. In conclusion, managing and mitigating H_2_S contamination is crucial for maintaining the SOFCs performance and lifespan, as even trace amounts can lead to significant efficiency losses and operational challenges.^136,137^

### 6.2 Nitrogenous Compounds

The effect of pure N_2_ gas on SOFCs has been scarcely studied in the literature, primarily because it is considered inert to the device and system components. Fuel dilution is one of the most concerning impacts, as it can lower the power generated by the cells.^138,139^ Additional effects of N_2_ addition to H_2_ in SOFCs include reduced fuel residence time and the blocking of active sites, which limits the availability of sites for charge transfer reactions.^138,140^

In a recent study, Hagen et al.^141^ compared the electrochemical performance of a commercial planar SOFC (Elcogen) operating with 3H_2_+N_2_ and 60%NH_3_ fed directly into the cell. They reported OCV degradation of 2.9% and 5.6%, respectively, at 700 °C. Additionally, Ni mesh current collectors in the NH_3_-powered SOFC exhibited nitridation and cracking, which may contribute to increased ohmic resistance during operation. This analysis supports the hypothesis that pure N_2_ gas alone does not significantly affect the cell. In another detailed study on the impact of N_2_ addition to H_2_, Li et al.^140^ demonstrated that N_2_ causes a severe increase in total SOFC resistance. When the H_2_:N_2_ ratio changed from 1:0 to 1:5, the total resistance increased by 3.5 times. Furthermore, the researchers found that while polarization resistance increased significantly, the rise in mass transport resistance was even more pronounced. This finding reinforces the hypothesis that N_2_ may impair fuel residence time or occupy active reaction sites, thereby hindering electrochemical processes.

However, the main concern regarding N_2_ in SOFCs arises when it originates from biogas and is converted into NH_3_ during catalytic reforming. Although this conversion is less common under the low-pressure conditions typical of catalytic reforming, NH_3_ can be further reduced to form hydrogen cyanide (HCN).

In this scenario, NH_3_ and HCN can be formed from the reaction of N_2_ with CH_4_. In a secondary reaction step, in the presence of HCN, it reacts with the produced H_2_ to produce additional NH_3_. Thus, the syngas obtained from biogas/biomethane, which contains N_2_, can also contain NH_3_. In contrast to N_2_, which is inert in SOFCs, NH_3_ can exhibit negative effects through poisoning. The level of N-contamination is heavily influenced by the type of biogas used as feedstock for reforming and SOFCs. NH_3_ is the predominant form of N_2_ contamination, which also leads to nitrogen oxide (NO_*x*_) emissions.^103^ There is controversy in the literature about the effect of N_2_ compounds on SOFCs. Some authors claim that there are no harmful effects, while others report that there are. The fact is that the influence of N_2_ and/or NH_3_ is dependent on the SOFC temperature operation.^142^ The dissociation of NH_3_ can proceed via two primary pathways.^143,144^ The first involves the thermal decomposition of a NH_3_ into N_2_ and H_2_, followed by the electro-oxidation of the generated H_2_ (eqs 12–13). The second pathway corresponds to the direct electro-oxidation of NH_3_ at the anode TPBs active sites (eqs 14):

14 $$2NH_{3}\to 3H_{2}+N_{2},\left(\Delta H>0,\;\mathrm{endothermic}\right)$$

15 $$H_{2}+O^{2-}\to H_{2}O+2e^{-}$$

16 $$NH_{3}+3O^{2-}\to N_{2}+3H_{2}O+6e^{-}$$

Furthermore, even under conditions where NH_3_ is thermodynamically favored at high temperatures, the presence of NH_3_ derived from biogas reactions can induce parallel degradation mechanisms.^145^ Specifically, adsorbed nitrogen intermediates (e.g., N-adsorbed) may accumulate on Ni surfaces instead of desorbing as N_2_, leading to the formation of Ni nitride (Ni_3_N), as eq 15 shows, or reactive gaseous species like NO in localized oxidizing zones.^146^ This phenomenon is particularly critical in systems operating at a lower temperature range (600–700 °C), where the kinetics of N-adsorbed desorption are slower, facilitating their accumulation and subsequent side reactions. While the H_2_ produced from NH_3_ decomposition sustains SOFC operation, residual N-adsorbed contamination can gradually reduce the density of active Ni sites at the TPBs, especially in biogas-fed systems where the composition of N_2_ (and its derivatives) varies significantly. The Ni_3_N formed is unstable and can react with H_2_ to produce NH_3_ and metallic Ni (eqs 16–17).^146^ This reaction may occur due to temperature fluctuations and can nucleate several microcracks at the SOFC anode, potentially leading to their propagation and structural degradation.^145,146^ Studies suggest, in addition to temperature optimization, that protective catalytic layers or reformate gas purification may be necessary to mitigate these effects without compromising system efficiency.

17 $$3Ni+NH_{3}\to Ni_{3}N+H_{2}$$

18 $$3H_{2}+2Ni_{3}N\to 2NH_{3}+6Ni$$

19 $$2Ni_{3}N\to N_{2}+6Ni$$

In summary, SOFCs are powered by NH_3_ in temperatures higher than 700 °C, they undergo thermal decomposition into N_2_ and H_2_, thus increasing the H_2_ concentration. H_2_ then participates in the same reaction processes as other gas fuels, producing energy. Thus, the nitrogenous compounds can also prevent coking.^147^ However, at lower temperatures, the anodes catalytic activity for NH_3_ decomposition decreased. Additionally, undecomposed NH_3_ may competitively occupy the TPB active sites for H_2_ adsorption. In short, this means that at high temperatures (above 700 °C), there is a positive effect from contaminant gases (i.e., N_2_ and NH_3_). However, under mild SOFC operating conditions (500–700 °C), these molecules start to compete for the active sites at the TPBs, resulting in reduced electrochemical activity and a drop in voltage output.^148^

One such case was studied by Yang et al.,^146^ where they employed an anode-supported SOFC based on Ni/YSZ anode powered by NH_3_ to investigate the influence of fuel exposure. The authors identified a phenomenon known as nitridation, in which a portion of the Ni in the anode is converted into Ni_3_N. Figure 8a shows the SEM image of the Ni/YSZ anode before NH_3_ exposure, showing a smooth surface morphology of the Ni particles. This smoothness indicates a well-sintered and percolated structure, very important for maintaining good electrical contact and lower resistances. In contrast, Figure 8b shows the Ni particles surface after 24 h of exposure to NH_3_ at 600 °C. The surface roughening and the formation of pores, smaller than 100 nm in diameter, suggest that the nitriding process has reshaped the microstructure. This roughening could weaken the contacts between Ni and YSZ particles, potentially increasing ohmic resistance and reducing cell performance. Moreover, the authors present EDX spectra confirming the presence of N_2_ on the Ni surface after nitriding. EDX spectra suggest that the nitriding process is temperature-dependent, which could affect the anode stability when operating with NH_3_. The XRD pattern in Figure 8c shows crystallographic changes in the Ni after NH_3_ exposure, with new peaks corresponding to Ni nitride phases, indicating nitriding. This change may impact the electrochemical properties of the anode, as Ni nitride differs from metallic Ni in terms of conductivity and catalytic behavior. Understanding these changes is essential for optimizing the anode material for NH_3_-fueled SOFCs. The OCV measurements over time for the anode fueled with H_2_ and NH_3_ during thermal cycling (Figure 8d) highlight the stability of the cell under different fuel conditions. The OCV for the NH_3_-fueled cell shows fluctuations, suggesting possible instability or degradation of the anode material over time. In contrast, the H_2_-fueled cell exhibits more stable OCV, indicating H_2_ is less harmful to the anode integrity. Furthermore, the authors show the polarization curves of anode performance before and after the temperature cycling test. The shift in the curves indicates decreased performance after exposure to NH_3_, likely due to nitriding and the related microstructural changes observed in the SEM images. This performance degradation highlights the challenges of using NH_3_ as a fuel, reinforcing the need for further research to improve the stability and performance of the Ni/YSZ anode in NH_3_ environments. Figure 8e provides a schematic representation of the SOFC, showing cracks that may result from the nitriding process and thermal cycling. These cracks can lead to delamination and increased resistance, worsening performance degradation. This schematic depicts the chemical and mechanical stresses that can develop in SOFCs, especially under varying temperatures and fuel compositions. The combination of several characterization techniques provides a detailed understanding of the effects of NH_3_ exposure on the Ni/YSZ anode. These findings highlight the importance of addressing stability and performance challenges when using NH_3_ as a fuel in SOFCs. Future research should focus on optimizing the anode materials and operational conditions to reduce the effects of nitriding and improve the NH_3_-fueled SOFCs efficiency.

**Figure 8:**
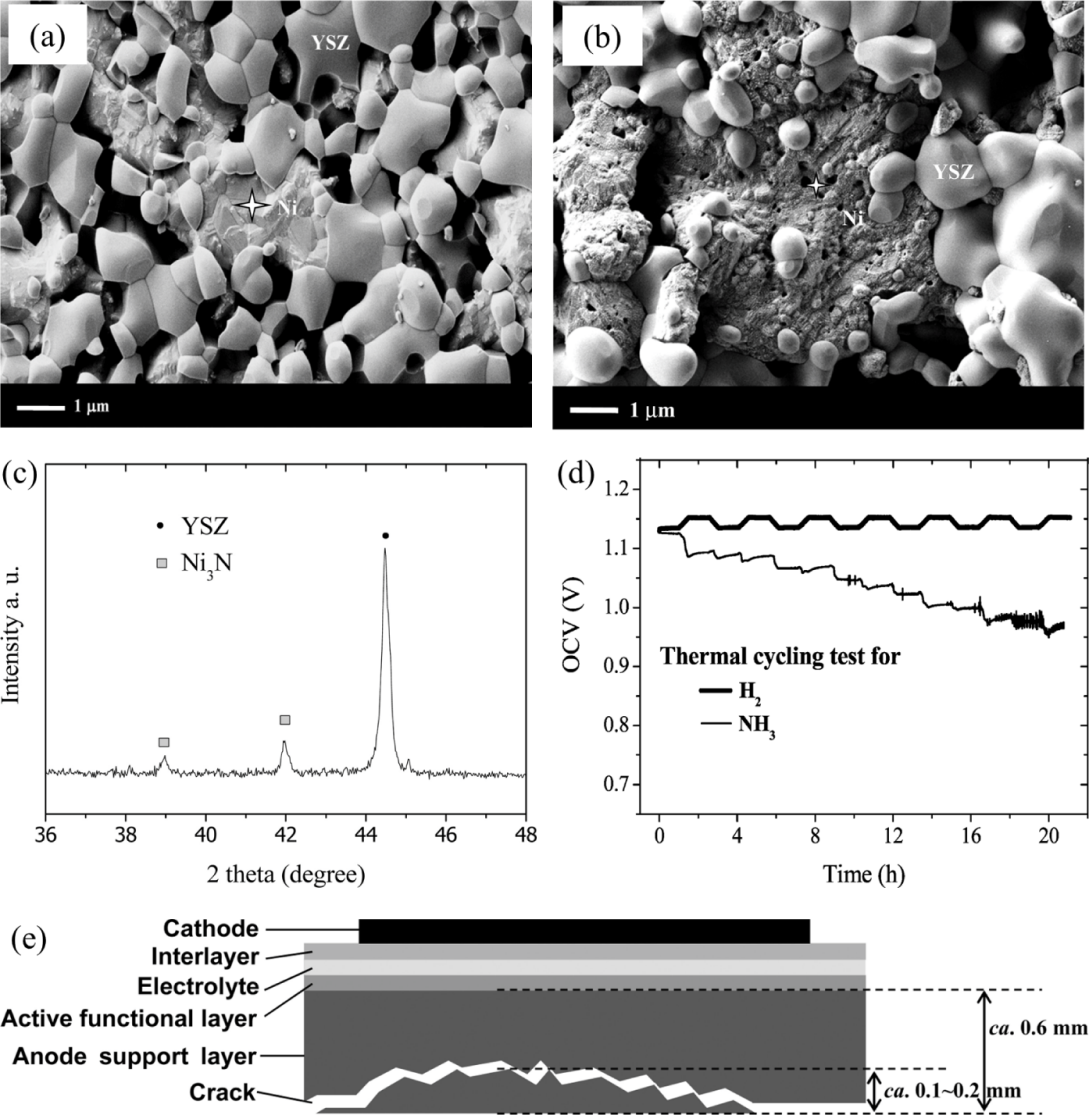
SEM images of Ni/YSZ cermet anode surface (a) before and (b) after exposure to NH_3_. (c) XRD pattern of Ni after being exposed to NH_3_. (d) OCV curves of the cell during thermal cycling tests fueled with and without NH_3_. (e) Scheme of the anode-supported SOFC after the temperature cycling test fueled by NH_3_. Reprinted with permission from.^146^ Copyright 2015, American Chemical Society.

In conclusion, although N_2_ and NH_3_ may initially appear to be inert gases merely occupying space in syngas and biomethane mixtures, their role in SOFCs is far from negligible. It can enhance H_2_ permeation, promote uniform fuel distribution, and optimize electrochemical processes. However, as previously discussed, N_2_ can also negatively impact the SOFC performance by diluting the fuel, thereby reducing power output. Despite the remaining issues related to contamination, NH_3_ holds significant potential as a direct fuel for SOFC systems, particularly when integrated with biodigester and reformer technologies. Ultimately, understanding and leveraging these effects can contribute to improved system designs and enhanced performance of FC technologies in energy transition applications.^149,150^

## 7 Perspectives and Conclusion

As discussed in this article, depending on the contaminants found in raw biogas, different techniques may be available for its treatment; however, none achieved complete removal 100% of H_2_S. Notably, biological desulfurization involving air injection often results in increasing N_2_ concentrations, leaving residual H_2_S levels between 10 and 50 ppm and N_2_ concentrations of approximately 5 to 8%. Both Ni-based catalysts employed in catalytic reforming and Ni-based anodes in SOFCs exhibit comparable sensitivities to contaminants. Even low concentrations of H_2_S (2–10 ppm) induce rapid catalyst poisoning and deactivation due to sulfur adsorption on active sites, with elevated concentrations (20–200 ppm) exacerbating catalyst sintering and irreversible formation of Ni sulfide phases. SOFC anodes demonstrate high susceptibility to sulfur poisoning, where trace amounts of H_2_S (as low as 0.05 ppm) cause significant degradation in electrochemical performance, manifesting as reduced current density and power output. Nitrogen, while largely inert during reforming, functions as a diluent that stabilizes reaction conditions but diminishes H yield at elevated concentrations. In SOFC systems, N_2_ contributes beneficially by facilitating NH_3_ decomposition, enhancing H permeation, and coking mitigating; however, low-temperature electro-oxidation of NH_3_ may lead to nitrate formation and subsequent anode degradation. To enhance sulfur tolerance, Ni-based catalysts and anodes can be engineered through the incorporation of sulfur-tolerant materials such as ceria-based supports or alloying with metals like molybdenum and tungsten to mitigate deactivation. Despite the potential formation of NH_3_ from N_2_ during reforming, its presence has been shown to facilitate H_2_ permeation and promote electrochemical stability and performance in SOFC systems.

Although challenges persist, the utilization of biogas and biomethane, particularly in fuel cell technologies, represents a promising pathway for advancing renewable energy systems and mitigating greenhouse gas emissions. Nonetheless, key barriers remain that must be overcome to realize the full potential of biogas in high-efficiency energy applications: a) future research should develop cost-effective, sulfur-tolerant or regenerable catalysts, such as perovskite or Pt/Rh composites, for CH_4_ reforming and SOFC anodes to overcome H_2_S-induced deactivation and enhance long-term durability; b) the impact of N_2_ in biogas must be further explored. Although inert under certain conditions, N_2_ can form NH_3_ during catalytic reforming, which negatively affects SOFCs performance, while developing catalysts and fuel cells capable of managing nitrogenous impurities; c) integrating biogas reforming with SOFCs for CHP enables efficient waste-to-energy conversion, enhances energy efficiency, and supports decentralized power generation, thereby accelerating biogas mainstream adoption.

Looking forward, the continued development of cleaner, more efficient biogas utilization technologies will support global efforts to transition to low-carbon energy systems, helping to mitigate climate change and foster energy independence. For this reason, this work supports the continued pursuit of technological innovations that maximize the potential of biogas for clean electricity generation, contributing to global efforts in reducing carbon emissions and fostering a sustainable energy future.

## Supplementary Material



## Notes

Tables S1–S3: alternative technologies for H_2_S removal processes; summary of substrates and parameters for H_2_S using micro-aeration; operating conditions and process efficiencies to conversion CH_4_/CO_2_ and molar ratio of H_2_/CO in the syngas (PDF)

## References

[1] Afotey B., Sarpong G. T. (2023). Estimation of Biogas Production Potential and Greenhouse
Gas Emissions Reduction for Sustainable Energy Management Using Intelligent
Computing Technique. Measurement: Sensors.

[2] de
Oliveira J. P. J., Antunes F. C., Dias T., Cesar R., Silva G. G., Hunt J., Doubek G., Zanin H. (2025). Porous Metal
Substrates for Solid Oxide Fuel Cells: Manufacturing Techniques and
Future Perspectives. Ceram. Int..

[3] Liu Y., Jia L., Chi B., Pu J., Li J. (2019). In Situ Exsolved Ni-Decorated
Ba­(Ce 0.9 Y 0.1) 0.8 Ni 0.2 O 3−δ Perovskite as Carbon-Resistant
Composite Anode for Hydrocarbon-Fueled Solid Oxide Fuel Cells. ACS Omega.

[4] Ma S., Hu X., Zhao Y., Wang X., Dong C. (2021). Design and Evaluation
of a Metal-Supported Solid Oxide Fuel Cell Vehicle Power System with
Bioethanol Onboard Reforming. ACS Omega.

[5] Antunes F. C., de Oliveira J. P. J., de Abreu R. S., Dias T., Brandão B. B. N. S., Gonçalves J. M., Ribeiro J., Hunt J., Zanin H., Doubek G. (2025). Reviewing Metal Supported Solid Oxide
Fuel Cells for Efficient Electricity Generation with Biofuels for
Mobility. J. Energy Chem..

[6] Pramuanjaroenkij A., Kakaç S. (2023). The Fuel Cell
Electric Vehicles: The Highlight Review. Int.
J. Hydrogen Energy.

[7] Fu S., Ye L., Shen F., He Y. (2023). Efficiency Maximization of a Direct
Internal Reforming Solid Oxide Fuel Cell in a Two-Layer Self-Optimizing
Control Structure. ACS Omega.

[8] Di
Costanzo N., Di Capua F., Cesaro A., Carraturo F., Salamone M., Guida M., Esposito G., Giordano A. (2024). Headspace
Micro-Oxygenation as a Strategy for Efficient Biogas Desulfurization
and Biomethane Generation in a Centralized Sewage Sludge Digestion
Plant. Biomass Bioenergy.

[9] Xu J., Wang X., Sun S., Zhao Y., Hu C. (2017). Effects of
Influent C/N Ratios and Treatment Technologies on Integral Biogas
Upgrading and Pollutants Removal from Synthetic Domestic Sewage. Sci. Rep..

[10] Atelge M. R., Senol H., Djaafri M., Hansu T. A., Krisa D., Atabani A., Eskicioglu C., MuratçobanoĞlu H., Unalan S., Kalloum S., Azbar N., Kıvrak H. D. (2021). A Critical
Overview of the State-of-the-Art Methods for Biogas Purification and
Utilization Processes. Sustainability.

[11] Werkneh A. A. (2022). Biogas
Impurities: Environmental and Health Implications, Removal Technologies
and Future Perspectives. Heliyon.

[12] Khan, M. U. ; Sarwar, A. ; Dutta, N. ; Arslan, M. The Biogas Use. In Biogas Plants; Wiley, 2024, pp. 117–140. 10.1002/9781119863946.ch6.

[13] Jha N. K., Lohani S. P., Khatiwada D., Pradhan P., Shakya S. R. (2024). Assessing
Greenhouse Gas Emissions and Decarbonization Potential of Household
Biogas Plant: Nepal’s Case Study. Energy
Sustainable Dev..

[14] Le
Pera A., Sellaro M., Pellegrino C., Limonti C., Siciliano A. (2024). Combined Pre-Treatment
Technologies for Cleaning Biogas before Its Upgrading to Biomethane:
An Italian Full-Scale Anaerobic Digester Case Study. Appl. Sci..

[15] Mulu E., M’Arimi M. M., Ramkat R. C. (2021). A Review of Recent Developments in
Application of Low Cost Natural Materials in Purification and Upgrade
of Biogas. Renewable Sustainable Energy Rev..

[16] Vikrant K., Kailasa S. K., Tsang D. C. W., Lee S. S., Kumar P., Giri B. S., Singh R. S., Kim K.-H. (2018). Biofiltration of
Hydrogen Sulfide: Trends and Challenges. J.
Cleaner Prod..

[17] González-Cortés, J. J. ; Almenglo, F. ; Ramírez, M. Biogas Purification Upgrading and Utilization: Focusing on Biological Systems. In Generation of Energy from Municipal Solid Waste; Springer Nature: Cham, Switzerland, 2024; pp. 281–312. DOI: 10.1007/978-3-031-74334-4_13.

[18] Nagendranatha
Reddy C., Bae S., Min B. (2019). Biological Removal
of H2S Gas in a Semi-Pilot Scale Biotrickling Filter: Optimization
of Various Parameters for Efficient Removal at High Loading Rates
and Low PH Conditions. Bioresour. Technol..

[19] Li X., Jiang X., Zhou Q., Jiang W. (2016). Effect of S/N Ratio
on the Removal of Hydrogen Sulfide from Biogas in Anoxic Bioreactors. Appl. Biochem. Biotechnol..

[20] Almenglo F., González-Cortés J. J., Ramírez M., Cantero D. (2023). Recent Advances in Biological Technologies
for Anoxic
Biogas Desulfurization. Chemosphere.

[21] Jepleting A., Mecha A. C., Sombei D., Moraa D., Chollom M. N. (2025). Potential
of Low-Cost Materials for Biogas Purification, a Review of Recent
Developments. Renewable Sustainable Energy Rev..

[22] Cano P. I., Colón J., Ramírez M., Lafuente J., Gabriel D., Cantero D. (2018). Life Cycle
Assessment of Different Physical-Chemical
and Biological Technologies for Biogas Desulfurization in Sewage Treatment
Plants. J. Cleaner Prod..

[23] Jiang X., Wu J., Jin Z., Yang S., Shen L. (2020). Enhancing the Removal
of H2S from Biogas through Refluxing of Outlet Gas in Biological Bubble-Column. Bioresour. Technol..

[24] Valle A., Fernández M., Ramírez M., Rovira R., Gabriel D., Cantero D. (2018). A Comparative Study of Eubacterial Communities by PCR-DGGE
Fingerprints in Anoxic and Aerobic Biotrickling Filters Used for Biogas
Desulfurization. Bioprocess Biosyst. Eng..

[25] Marín D., Vega M., Lebrero R., Muñoz R. (2020). Optimization
of a Chemical Scrubbing Process Based on a Fe-EDTA-Carbonate Based
Solvent for the Simultaneous Removal of CO2 and H2S from Biogas. J. Water Process. Eng..

[26] Schiavon
Maia D. C., Niklevicz R. R., Arioli R., Frare L. M., Arroyo P. A., Gimenes M. L., Pereira N. C. (2017). Removal of H 2 S
and CO 2 from Biogas in Bench Scale and the Pilot Scale Using a Regenerable
Fe-EDTA Solution. Renew. Energy.

[27] Wubs H. J., Beenackers A. A. C. M. (1993). Kinetics
of the Oxidation of Ferrous Chelates of EDTA
and HEDTA in Aqueous Solution. Ind. Eng. Chem.
Res..

[28] Das J., Nolan S., Lens P. N. L. (2022). Simultaneous Removal of H2S and NH3
from Raw Biogas in Hollow Fibre Membrane Bioreactors. Environ. Technol. Innov..

[29] Dean P. A., Mizrahi Rodriguez K., Guo S., Roy N., Swager T. M., Smith Z. P. (2024). Elucidating the Role of Micropore-Generating
Backbone
Motifs and Amine Functionality on H2S, CO2, CH4 and N2 Sorption. J. Membr. Sci..

[30] Chan Y. H., Lock S. S. M., Wong M. K., Yiin C. L., Loy A. C. M., Cheah K. W., Chai S. Y. W., Li C., How B. S., Chin B. L. F., Chan Z. P., Lam S. S. (2022). A State-of-the-Art
Review on Capture and Separation of Hazardous Hydrogen Sulfide (H2S):
Recent Advances, Challenges and Outlook. Environ.
Pollut..

[31] Da
Conceicao M., Nemetz L., Rivero J., Hornbostel K., Lipscomb G. (2023). Gas Separation Membrane Module Modeling: A Comprehensive
Review. Membranes.

[32] Brunetti A., Lei L., Avruscio E., Karousos D. S., Lindbråthen A., Kouvelos E. P., He X., Favvas E. P., Barbieri G. (2022). Long-Term
Performance of Highly Selective Carbon Hollow Fiber Membranes for
Biogas Upgrading in the Presence of H2S and Water Vapor. Chem. Eng. J..

[33] Baker R. W., Lokhandwala K. (2008). Natural Gas Processing with Membranes: An Overview. Ind. Eng. Chem. Res..

[34] Roozitalab A., Hamidavi F., Kargari A. (2023). A Review of
Membrane Material for
Biogas and Natural Gas Upgrading. Gas Sci. Eng..

[35] Oliveira L. G., Cremonez P. A., Machado B., da Silva E. S., Silva F. E. B., Corrêa G. C. G., Lopez T. F. M., Alves H. J. (2023). Updates
on Biogas Enrichment and Purification Methods: A Review. Can. J. Chem. Eng..

[36] Scheufele F. B., da Silva E. S., Cazula B. B., Marins D. S., Sequinel R., Borba C. E., Patuzzo G. S., Lopez T. F. M., Alves H. J. (2021). Mathematical
Modeling of Low-Pressure H2S Adsorption by Babassu Biochar in Fixed
Bed Column. J. Environ. Chem. Eng..

[37] Balsamo M., Cimino S., de Falco G., Erto A., Lisi L. (2016). ZnO-CuO Supported
on Activated Carbon for H2S Removal at Room Temperature. Chem. Eng. J..

[38] Kim H., Ko K.-J., Mofarahi M., Kim K.-M., Lee C.-H. (2023). Adsorption
Behavior and Mechanism of Ultra-Low Concentration Sulfur Compounds
in Natural Gas on Cu-Impregnated Activated Carbon. Chem. Eng. J..

[39] Liu C., Zhang R., Wei S., Wang J., Liu Y., Li M., Liu R. (2015). Selective
Removal of H 2 S from Biogas Using a Regenerable
Hybrid TiO 2/Zeolite Composite. Fuel.

[40] Bahraminia S., Anbia M., Koohsaryan E. (2020). Hydrogen Sulfide
Removal from Biogas
Using Ion-Exchanged Nanostructured NaA Zeolite for Fueling Solid Oxide
Fuel Cells. Int. J. Hydrogen Energy.

[41] Ozekmekci M., Salkic G., Fellah M. F. (2015). Use of Zeolites for the Removal of
H 2 S: A Mini-Review. Fuel Process. Technol..

[42] Liu X., Wang R. (2017). Effective Removal of
Hydrogen Sulfide Using 4A Molecular Sieve Zeolite
Synthesized from Attapulgite. J. Hazard. Mater..

[43] Coppola G., Papurello D. (2019). Biogas Cleaning:
Activated Carbon Regeneration for
H2S Removal. Clean Technol..

[44] Abdirakhimov M., Al-Rashed M. H., Wójcik J. (2024). Hydrogen Sulfide Adsorption from
Natural Gas Using Silver-Modified 13X Molecular Sieve. Materials.

[45] Díaz I., Ramos I., Fdz-Polanco M. (2015). Economic Analysis of Microaerobic
Removal of H2S from Biogas in Full-Scale Sludge Digesters. Bioresour. Technol..

[46] Andreides M., Pokorná-Krayzelová L., Bartáček J. (2024). Importance
of Digester’s Headspace Geometry for the Efficient H2S Removal
through Microaeration; Experimental and Simulation Study. Fuel.

[47] Krayzelova L., Bartacek J., Kolesarova N., Jenicek P. (2014). Microaeration for Hydrogen
Sulfide Removal in UASB Reactor. Bioresour.
Technol..

[48] Jeníček P., Horejš J., Pokorná-Krayzelová L., Bindzar J., Bartáček J. (2017). Simple Biogas Desulfurization
by Microaeration – Full Scale Experience. Anaerobe.

[49] Krayzelova L., Bartacek J., Díaz I., Jeison D., Volcke E. I. P., Jenicek P. (2015). Microaeration for Hydrogen Sulfide Removal during Anaerobic
Treatment: A Review. Rev. Environ. Sci. Biotechnol..

[50] Chen Q., Wu W., Qi D., Ding Y., Zhao Z. (2020). Review on Microaeration-Based
Anaerobic Digestion: State of the Art, Challenges, and Prospectives. Sci. Total Environ..

[51] Fu S., Lian S., Angelidaki I., Guo R. (2023). Micro-Aeration: An
Attractive Strategy to Facilitate Anaerobic Digestion. Trends Biotechnol..

[52] Azizi S. M. M., Zakaria B. S., Haffiez N., Niknejad P., Dhar B. R. (2022). A Critical
Review of Prospects and Operational Challenges of Microaeration and
Iron Dosing for In-Situ Biogas Desulfurization. Bioresour. Technol. Rep..

[53] Kraakman N. J. R., Diaz I., Fdz-Polanco M., Muñoz R. (2023). Large-Scale
Micro-Aerobic Digestion Studies at Municipal Water Resource Recovery
Facilities for Process-Integrated Biogas Desulfurization. J. Water Process. Eng..

[54] Díaz I., Lopes A. C., Pérez S. I., Fdz-Polanco M. (2010). Performance
Evaluation of Oxygen, Air and Nitrate for the Microaerobic Removal
of Hydrogen Sulphide in Biogas from Sludge Digestion. Bioresour. Technol..

[55] Yi X.-H., Wan J., Ma Y., Wang Y., Guan Z., Jing D.-D. (2017). Structure
and Succession of Bacterial Communities of the Granular Sludge during
the Initial Stage of the Simultaneous Denitrification and Methanogenesis
Process. Water, Air, Soil Pollut..

[56] Curiel-Esparza J., Reyes-Medina M., Martin-Utrillas M., Martinez-Garcia M. P., Canto-Perello J. (2019). Collaborative
Elicitation to Select a Sustainable Biogas
Desulfurization Technique for Landfills. J.
Cleaner Prod..

[57] Tápparo D. C., Cândido D., Steinmetz R. L. R., Etzkorn C., Do Amaral A. C., Antes F. G., Kunz A. (2021). Swine Manure
Biogas Production Improvement
Using Pre-Treatment Strategies: Lab-Scale Studies and Full-Scale Application. Bioresour. Technol. Rep..

[58] Khadir A., Nakhla G., Karki R., Raskin L., Muller C., Guevarra K., Summers A., Pierce L., Shahbaz P., Bell K., Skerlos S., Bronstad E. (2025). Micro-Aeration
for
Hydrogen Sulfide Reduction in Full-Scale Anaerobic Digesters with
Limited Headspace: Performance and Sulfide Reduction Kinetics. Process Saf. Environ. Prot..

[59] Faheem H. H., Tanveer H. U., Abbas Syed Z., Maqbool F. (2021). Comparative study of
conventional steam-methane-reforming (SMR) and auto-thermal-reforming
(ATR) with their hybrid sorption enhanced (SE-SMR & SE-ATR) and
environmentally benign process models for the hydrogen production. Fuel.

[60] Garcia-Villalva R., Biset-Peiró M., Alarcón A., Bacariza C., Murcia-López S., Guilera J. (2024). Comparison of Methane Reforming Routes for Hydrogen
Production Using Dielectric Barrier Discharge Plasma-Catalysis. Int. J. Hydrogen Energy.

[61] Wang Z., Burra K. G., Zhang M., Li X., He X., Lei T., Gupta A. K. (2020). Syngas Evolution
and Energy Efficiency in CO2-Assisted
Gasification of Pine Bark. Appl. Energy.

[62] Sonal, Ahmad E., Upadhyayula S., Pant K. K. (2019). Biomass-Derived CO2 Rich Syngas Conversion to Higher
Hydrocarbon via Fischer–Tropsch Process over Fe–Co Bimetallic
Catalyst. Int. J. Hydrogen Energy.

[63] Radenahmad N., Azad A. T., Saghir M., Taweekun J., Bakar M. S. A., Reza M. S., Azad A. K. (2020). A Review on Biomass Derived Syngas
for SOFC Based Combined Heat and Power Application. Renewable Sustainable Energy Rev..

[64] Su B., Wang Y., Xu Z., Han W., Jin H., Wang H. (2022). Novel Ways for Hydrogen Production Based on Methane Steam and Dry
Reforming Integrated with Carbon Capture. Energy
Convers. Manage..

[65] Fernandes D. J., Ferreira A. F., Fernandes E. C. (2023). Biogas and Biomethane Production
Potential via Anaerobic Digestion of Manure: A Case Study of Portugal. Renewable Sustainable Energy Rev..

[66] Cabello A., Mendiara T., Izquierdo M. T., de Diego L., Abad A. (2024). Conversion
of Dry Biogas in a Chemical Looping Reforming Unit: Performance of
Fe and FeMn-Based Oxygen Carriers. Int. J. Hydrogen
Energy.

[67] Alves H. J., Bley Junior C., Niklevicz R. R., Frigo E. P., Frigo M. S., Coimbra-Araújo C. H. (2013). Overview of Hydrogen Production Technologies
from Biogas and the Applications in Fuel Cells. Int. J. Hydrogen Energy.

[68] Spath, P. L. ; Mann, M. K. Life Cycle Assessment of Hydrogen Production via Natural Gas Steam Reforming; NREL: Golden, CO, United States, 2000. 10.2172/764485.

[69] Oni A. O., Anaya K., Giwa T., Di Lullo G., Kumar A. (2022). Comparative
Assessment of Blue Hydrogen from Steam Methane Reforming, Autothermal
Reforming, and Natural Gas Decomposition Technologies for Natural
Gas-Producing Regions. Energy Convers. Manage..

[70] Nguyen D. L. T., Vy Tran A., Vo D.-V. N., Tran Nguyen H., Rajamohan N., Trinh T. H., Nguyen T. L., Le Q. V., Nguyen T. M. (2024). Methane Dry Reforming: A Catalyst
Challenge Awaits. J. Ind. Eng. Chem..

[71] Zhao K., He F., Huang Z., Wei G., Zheng A., Li H., Zhao Z. (2016). CaO/MgO Modified Perovskite
Type Oxides for Chemical-Looping Steam
Reforming of Methane. J. Fuel Chem. Technol..

[72] Zhao K., Zhang R., Gao Y., Lin Y., Liu A., Wang X., Zheng A., Huang Z., Zhao Z. (2022). High Syngas
Selectivity and near Pure Hydrogen Production in Perovskite Oxygen
Carriers for Chemical Looping Steam Methane Reforming. Fuel Process. Technol..

[73] Wang F., Chen S., Chen S., Du J., Duan L., Xiang W. (2023). Double Adjustment of Ni and Co in CeO2/La2Ni2-XCoxO6 Double Perovskite
Type Oxygen Carriers for Chemical Looping Steam Methane Reforming. Chem. Eng. J..

[74] Pawar V., Appari S., Monder D. S., Janardhanan V. M. (2017). Study of
the Combined Deactivation Due to Sulfur Poisoning and Carbon Deposition
during Biogas Dry Reforming on Supported Ni Catalyst. Ind. Eng. Chem. Res..

[75] Celik N. K., Yasyerli S., Arbag H., Tasdemir H. M., Yasyerli N. (2025). Regenerable
Nickel Catalysts Strengthened against H2S Poisoning in Dry Reforming
of Methane. Fuel.

[76] Jablonski W. S., Villano S. M., Dean A. M. (2015). A Comparison of
H2S, SO2, and COS
Poisoning on Ni/YSZ and Ni/K2O-CaAl2O4 during Methane Steam and Dry
Reforming. Appl. Catal., A.

[77] Akansu H., Arbag H., Tasdemir H. M., Yasyerli S., Yasyerli N., Dogu G. (2022). Nickel-Based Alumina
Supported Catalysts for Dry Reforming of Biogas
in the Absence and the Presence of H2S: Effect of Manganese Incorporation. Catal. Today.

[78] Zheng J., Impeng S., Liu J., Deng J., Zhang D. (2024). Mo Promoting
Ni-Based Catalysts Confined by Halloysite Nanotubes for Dry Reforming
of Methane: Insight of Coking and H2S Poisoning Resistance. Appl. Catal., B.

[79] Das S., Lim K. H., Gani T. Z. H., Aksari S., Kawi S. (2023). Bi-Functional
CeO2 Coated NiCo-MgAl Core-Shell Catalyst with High Activity and Resistance
to Coke and H2S Poisoning in Methane Dry Reforming. Appl. Catal., B.

[80] Capa A., González-Vázquez M. P., Chen D., Rubiera F., Pevida C., Gil M. V. (2023). Effect of H2S on Biogas Sorption
Enhanced Steam Reforming Using a Pd/Ni-Co Catalyst and Dolomite as
a Sorbent. Chem. Eng. J..

[81] Hernandez A. D., Kaisalo N., Simell P., Scarsella M. (2019). Effect of
H2S and Thiophene on the Steam Reforming Activity of Nickel and Rhodium
Catalysts in a Simulated Coke Oven Gas Stream. Appl. Catal., B.

[82] Vecino-Mantilla S., Simon P., Huvé M., Gauthier G., Gauthier-Maradei P. (2020). Methane Steam
Reforming in Water-Deficient Conditions on a New Ni-Exsolved Ruddlesden-Popper
Manganite: Coke Formation and H2S Poisoning. Int. J. Hydrogen Energy.

[83] Mota N., Ismagilov I. Z., Matus E. V., Kuznetsov V. V., Kerzhentsev M. A., Ismagilov Z. R., Navarro R. M., Fierro J. L. G. (2016). Hydrogen
Production by Autothermal Reforming of Methane over Lanthanum Chromites
Modified with Ru and Sr. Int. J. Hydrogen Energy.

[84] Wachter P., Gaber C., Raic J., Demuth M., Hochenauer C. (2021). Experimental
Investigation on H2S and SO2 Sulphur Poisoning and Regeneration of
a Commercially Available Ni-Catalyst during Methane Tri-Reforming. Int. J. Hydrogen Energy.

[85] Nirmal
Kumar S., Appari S., Kuncharam B. V. R. (2021). Techniques
for Overcoming Sulfur Poisoning of Catalyst Employed in Hydrocarbon
Reforming. Catal. Surv. Asia.

[86] Poggio-Fraccari E., Herrera C., Larrubia M. A., Alemany L., Laborde M., Mariño F. (2024). Study of a
Catalytic Technology for Syngas/H2 Production
from Raw Biogas Self-Reforming in Presence of Sulphur. Int. J. Hydrogen Energy.

[87] Chelvam K., Hanafiah M. M., Alkhatib I. I. I., Ali S. M., Vega L. F. (2025). Life Cycle
Assessment on the Role of H2S-Based Hydrogen via H2S-Methane Reforming
for the Production of Sustainable Fuels. Sci.
Total Environ..

[88] Gao Y., Aihemaiti A., Jiang J., Meng Y., Ju T., Han S., Chen X., Liu J. (2020). Inspection over Carbon Deposition
Features of Various Nickel Catalysts during Simulated Biogas Dry Reforming. J. Cleaner Prod..

[89] Al-Fatesh A. S., Atia H., Abu-Dahrieh J. K., Ibrahim A. A., Eckelt R., Armbruster U., Abasaeed A. E., Fakeeha A. H. (2019). Hydrogen Production
from CH4 Dry Reforming over Sc Promoted Ni/MCM-41. Int. J. Hydrogen Energy.

[90] Shen Z., Nabavi S. A., Clough P. T. (2024). Intrinsic
Kinetics of Steam Methane
Reforming over a Monolithic Nickel Catalyst in a Fixed Bed Reactor
System. Chem. Eng. Sci..

[91] Watanabe F., Kaburaki I., Shimoda N., Satokawa S. (2016). Influence
of Nitrogen
Impurity for Steam Methane Reforming over Noble Metal Catalysts. Fuel Process. Technol..

[92] Adiya S. G. Z. I., Dupont V., Mahmud T. (2017). Effect of Hydrocarbon
Fractions,
N2 and CO2 in Feed Gas on Hydrogen Production Using Sorption Enhanced
Steam Reforming: Thermodynamic Analysis. Int.
J. Hydrogen Energy.

[93] Bolívar
Caballero J. J., Zaini I. N., Yang W. (2022). Reforming Processes
for Syngas Production: A Mini-Review on the Current Status, Challenges,
and Prospects for Biomass Conversion to Fuels. Appl. Energy Combust. Sci..

[94] Brus G., Komatsu Y., Kimijima S., Szmyd J. (2012). An Analysis of Biogas
Reforming Process on Ni/YSZ and Ni/SDC Catalysts. Int. J. Thermodyn..

[95] Cao J.-P., Shi P., Zhao X.-Y., Wei X.-Y., Takarada T. (2014). Catalytic Reforming
of Volatiles and Nitrogen Compounds from Sewage Sludge Pyrolysis to
Clean Hydrogen and Synthetic Gas over a Nickel Catalyst. Fuel Process. Technol..

[96] Chein R. Y., Chen Y. C., Yu C. T., Chung J. N. (2015). Thermodynamic
Analysis
of Dry Reforming of CH4 with CO2 at High Pressures. J. Nat. Gas Sci. Eng..

[97] Nanadegani F. S., Sunden B. (2023). Review of Exergy and Energy Analysis
of Fuel Cells. Int. J. Hydrogen Energy.

[98] Wasajja H., Lindeboom R. E. F., van Lier J. B., Aravind P. V. (2020). Techno-Economic
Review of Biogas Cleaning Technologies for Small Scale off-Grid Solid
Oxide Fuel Cell Applications. Fuel Process.
Technol..

[99] Hasanzadeh A., Chitsaz A., Mojaver P., Ghasemi A. (2021). Stand-Alone Gas Turbine
and Hybrid MCFC and SOFC-Gas Turbine Systems: Comparative Life Cycle
Cost, Environmental, and Energy Assessments. Energy Rep..

[100] Saadabadi S. A., Thallam Thattai A., Fan L., Lindeboom R. E. F., Spanjers H., Aravind P. V. (2019). Solid Oxide Fuel Cells Fuelled with
Biogas: Potential and Constraints. Renew. Energy.

[101] Lu P., Wei S., DU Z., Ma W., Ni S. (2024). Analysis and
Comparison of Multi-Physics Fields for Different Flow Field Configurations
in SOFC. Int. J. Heat Mass Transfer..

[102] Kunze-Liebhäuser, J. ; Paschos, O. ; Pethaiah, S. S. ; Stimming, U. Fuel Cell Comparison to Alternate Technologies. In Encyclopedia of Sustainability Science and Technology; Springer New York: New York, NY, 2017; pp. 1–16. DOI: 10.1007/978-1-4939-2493-6_157-3.

[103] Alaedini A. H., Tourani H. K., Saidi M. (2023). A Review of
Waste-to-Hydrogen
Conversion Technologies for Solid Oxide Fuel Cell (SOFC) Applications:
Aspect of Gasification Process and Catalyst Development. J. Environ. Manage..

[104] Papurello D., Lanzini A., Fiorilli S., Smeacetto F., Singh R., Santarelli M. (2016). Sulfur Poisoning in Ni-Anode Solid
Oxide Fuel Cells (SOFCs): Deactivation in Single Cells and a Stack. Chem. Eng. J..

[105] Sivasankar C., Baskaran S., Tamizmani M., Ramakrishna K. (2014). Lessons Learned and Lessons to Be Learned for Developing
Homogeneous Transition Metal Complexes Catalyzed Reduction of N2 to
Ammonia. J. Organomet. Chem..

[106] Chen H., Wang F., Wang W., Chen D., Li S.-D., Shao Z. (2016). H2S Poisoning Effect
and Ways to
Improve Sulfur Tolerance of Nickel Cermet Anodes Operating on Carbonaceous
Fuels. Appl. Energy.

[107] Sun S., Cheng Z. (2018). H 2 S Poisoning of
Proton Conducting Solid Oxide Fuel
Cell and Comparison with Conventional Oxide-Ion Conducting Solid Oxide
Fuel Cell. J. Electrochem. Soc..

[108] Rasmussen J. F. B., Hagen A. (2009). The Effect of H2S on the Performance
of Ni–YSZ Anodes in Solid Oxide Fuel Cells. J. Power Sources.

[109] Brightman E., Ivey D. G., Brett D. J. L., Brandon N. P. (2011). The Effect
of Current Density on H2S-Poisoning of Nickel-Based Solid Oxide Fuel
Cell Anodes. J. Power Sources.

[110] Lohsoontorn P., Brett D. J. L., Brandon N. P. (2008). The Effect of Fuel
Composition and Temperature on the Interaction of H2S with Nickel–Ceria
Anodes for Solid Oxide Fuel Cells. J. Power
Sources.

[111] Gong M., Liu X., Trembly J., Johnson C. (2007). Sulfur-Tolerant
Anode Materials for Solid Oxide Fuel Cell Application. J. Power Sources.

[112] Yokokawa H., Horita T., Yamaji K., Kishimoto H., Brito M. E. (2012). Degradation of SOFC Cell/Stack Performance
in Relation
to Materials Deterioration. J. Korean Ceram.
Soc..

[113] Marcantonio V., Del Zotto L., Ouweltjes J. P., Bocci E. (2022). Main Issues of the Impact of Tar, H2S, HCl and Alkali Metal from
Biomass-Gasification Derived Syngas on the SOFC Anode and the Related
Gas Cleaning Technologies for Feeding a SOFC System: A Review. Int. J. Hydrogen Energy.

[114] Vivet N., Chupin S., Estrade E., Piquero T., Pommier P. L., Rochais D., Bruneton E. (2011). 3D Microstructural
Characterization of a Solid Oxide Fuel Cell Anode Reconstructed by
Focused Ion Beam Tomography. J. Power Sources.

[115] Boldrin P., Ruiz-Trejo E., Mermelstein J., Bermúdez Menéndez J. M., Ramírez Reina T., Brandon N. P. (2016). Strategies for Carbon
and Sulfur Tolerant Solid Oxide
Fuel Cell Materials, Incorporating Lessons from Heterogeneous Catalysis. Chem. Rev..

[116] Kim J. H., Liu M., Chen Y., Murphy R., Choi Y., Liu Y., Liu M. (2021). Understanding the Impact
of Sulfur Poisoning on the Methane-Reforming Activity of a Solid Oxide
Fuel Cell Anode. ACS Catal..

[117] Mai Thi H. H., Rosman N., Sergent N., Pagnier T. (2017). Impedance
and Raman Spectroscopy Study of Effect of H 2 S on Ni-YSZ SOFC Anodes. Fuel Cells.

[118] Cheng Z., Abernathy H., Liu M. (2007). Raman Spectroscopy
of Nickel Sulfide Ni 3 S 2. J. Phys. Chem. C.

[119] Zarabi Golkhatmi S., Asghar M. I., Lund P. D. (2022). A Review on Solid
Oxide Fuel Cell Durability: Latest Progress, Mechanisms, and Study
Tools. Renewable Sustainable Energy Rev..

[120] Goodenough J. B., Huang Y.-H. (2007). Alternative Anode
Materials for Solid
Oxide Fuel Cells. J. Power Sources.

[121] Matsuzaki Y. (2000). The Poisoning Effect of Sulfur-Containing
Impurity
Gas on a SOFC Anode: Part I. Dependence on Temperature, Time, and
Impurity Concentration. Solid State Ionics.

[122] Roushanafshar M., Yan N., Chuang K. T., Luo J.-L. (2015). Electrochemical
Oxidation of Sour Natural Gas over La0.4Ce0.6O1.8–La0.4Sr0.6TiO3±δ
Anode in SOFC: A Mechanism Study of H2S Effects. Appl. Catal., B.

[123] da Silva A. L., Heck N. C. (2015). Thermodynamics of Sulfur Poisoning
in Solid Oxide Fuel Cells Revisited: The Effect of H2S Concentration,
Temperature, Current Density and Fuel Utilization. J. Power Sources.

[124] Kim K. I., Kim H. S., Kim H. S., Yun J. W. (2018). H2S Tolerance
Effects of Ce0.8Sm0.2O2– Modification on Sr0.92Y0.08Ti1–xNixO3–
Anode in Solid Oxide Fuel Cells. J. Ind. Eng.
Chem..

[125] Sapountzi F. M., Tsampas M. N., Zhao C., Boreave A., Retailleau L., Montinaro D., Vernoux P. (2015). Triode Operation for
Enhancing the Performance of H2S-Poisoned SOFCs Operated under CH4–H2O
Mixtures. Solid State Ionics.

[126] Hagen A., Rasmussen J. F. B., Thydén K. (2011). Durability
of Solid Oxide Fuel Cells Using Sulfur Containing Fuels. J. Power Sources.

[127] Yoshizumi T., Taniguchi S., Shiratori Y., Sasaki K. (2012). Sulfur Poisoning of SOFCs: Voltage
Oscillation and
Ni Oxidation. J. Electrochem. Soc..

[128] Wasajja H., Saadabadi S. A., Illathukandy B., Lindeboom R. E. F., van Lier J. B., Vellayani Aravind P. (2022). The Effect
of H 2 S on Internal Dry Reforming in Biogas Fuelled Solid Oxide Fuel
Cells. Energy Sci. Eng..

[129] Li J., Wei B., Yue X., Su C., Lü Z. (2020). Investigations
on Sulfur Poisoning Mechanisms of a Solid Oxide Fuel Cell with Niobium-Doped
Ferrate Perovskite Anode. Electrochim. Acta.

[130] Price R., Grolig J. G., Mai A., Irvine J. T. S. (2020). Evaluating
Sulfur-Tolerance of Metal/Ce0.80Gd0.20O1.90 Co-Impregnated La0.20Sr0.25Ca0.45TiO3
Anodes for Solid Oxide Fuel Cells. Solid State
Ionics.

[131] Azad A.-M., Duran M. J. (2007). Development of Ceria-Supported
Sulfur
Tolerant Nanocatalysts: Rh-Based Formulations. Appl. Catal., A.

[132] Cheng Z., Wang J.-H., Choi Y., Yang L., Lin M. C., Liu M. (2011). From Ni-YSZ to Sulfur-Tolerant Anode
Materials for SOFCs: Electrochemical Behavior, in Situ Characterization,
Modeling, and Future Perspectives. Energy Environ.
Sci..

[133] Sun S., Awadallah O., Cheng Z. (2018). Poisoning of Ni-Based Anode for Proton
Conducting SOFC by H2S, CO2, and H2O as Fuel Contaminants. J. Power Sources.

[134] Yang L., Wang S., Blinn K., Liu M., Liu Z., Cheng Z., Liu M. (2009). Enhanced Sulfur and
Coking Tolerance
of a Mixed Ion Conductor for SOFCs: BaZr 0.1 Ce 0.7 Y 0.2– *x* Yb *x* O 3−δ. Science.

[135] Kim J. H., Chern Z.-Y., Yoo S., DeGlee B., Wang J., Liu M. (2020). Unraveling the Mechanism
of Water-Mediated
Sulfur Tolerance via Operando Surface-Enhanced Raman Spectroscopy. ACS Appl. Mater. Interfaces.

[136] Cayan F. N., Zhi M., Pakalapati S. R., Celik I., Wu N., Gemmen R. (2008). Effects of Coal Syngas
Impurities on Anodes of Solid Oxide Fuel Cells. J. Power Sources.

[137] Corigliano O., Pagnotta L., Fragiacomo P. (2022). On the Technology
of Solid Oxide Fuel Cell (SOFC) Energy Systems for Stationary Power
Generation: A Review. Sustainability.

[138] Le Gal La Salle A., Ricoul F., Joubert O., Kerihuel A., Subrenat A. (2017). Electrochemical Study of a SOFC with
Various H 2 -CO-CH
4 -CO 2 -N 2 Gaseous Mixtures. Fuel Cells.

[139] Cinti G., Desideri U., Penchini D., Discepoli G. (2014). Experimental
Analysis of SOFC Fuelled by Ammonia. Fuel Cells.

[140] Li H., Wei W., Liu F., Xu X., Li Z., Liu Z. (2023). Identification of Internal
Polarization Dynamics for Solid Oxide
Fuel Cells Investigated by Electrochemical Impedance Spectroscopy
and Distribution of Relaxation Times. Energy.

[141] Hagen A., Caldogno R., Sun X. (2024). Direct Ammonia
SOFC
– A Potential Technology for Green Shipping. Fuel.

[142] Ud Din Z., Zainal Z. A. (2016). Biomass Integrated
Gasification–SOFC
Systems: Technology Overview. Renewable Sustainable
Energy Rev..

[143] Rathore S. S., Biswas S., Fini D., Kulkarni A. P., Giddey S. (2021). Direct Ammonia
Solid-Oxide Fuel Cells: A Review of
Progress and Prospects. Int. J. Hydrogen Energy.

[144] Wojcik A., Middleton H., Damopoulos I., Van Herle J. (2003). Ammonia as
a Fuel in Solid Oxide Fuel Cells. J. Power Sources.

[145] Oh S., Oh M. J., Hong J., Yoon K. J., Ji H.-I., Lee J.-H., Kang H., Son J.-W., Yang S. (2022). A Comprehensive
Investigation of Direct Ammonia-Fueled Thin-Film Solid-Oxide Fuel
Cells: Performance, Limitation, and Prospects. iScience.

[146] Yang J., Molouk A. F. S., Okanishi T., Muroyama H., Matsui T., Eguchi K. (2015). A Stability Study of Ni/Yttria-Stabilized
Zirconia Anode for Direct Ammonia Solid Oxide Fuel Cells. ACS Appl. Mater. Interfaces.

[147] Xu L., Dong F., Zhuang H., He W., Ni M., Feng S.-P., Lee P.-H. (2017). Energy Upcycle in
Anaerobic Treatment:
Ammonium, Methane, and Carbon Dioxide Reformation through a Hybrid
Electrodeionization–Solid Oxide Fuel Cell System. Energy Convers. Manage..

[148] Lin Y., Ran R., Guo Y., Zhou W., Cai R., Wang J., Shao Z. (2010). Proton-Conducting
Fuel Cells Operating
on Hydrogen, Ammonia and Hydrazine at Intermediate Temperatures. Int. J. Hydrogen Energy.

[149] Jang I., Carneiro S. A. J., Crawford J. O., Cho Y. J., Parvin S., Gonzalez-Casamachin D.
A., Baltrusaitis J., Lively R. P., Nikolla E. (2024). Electrocatalysis in Solid Oxide Fuel
Cells and Electrolyzers. Chem. Rev..

[150] Rahimipetroudi I., Omer A., Hwan Park S., Haeng Hur J., Won Lee D., Rashid K., Bok Yang J., Keun Dong S. (2024). Efficient 5 KW-Class Solid Oxide Fuel Cell (SOFC) Hotbox
Design with off Gas Integration for Power Generation. Appl. Therm. Eng..

