# Supplementary material for: A Brief Review of H2S and Nitrogen-Based Contaminants from Biogas: Effects on Reforming Catalysts and SOFC Anodes
Source: ACS Omega. 2025 Nov 18;10(47):56913–31. doi: 10.1021/acsomega.5c02399 (PMC12676356; doi:10.1021/acsomega.5c02399)
Supplement: Supplementary file 1 [file ao5c02399_si_001.pdf]

Supplementary Information

A Brief Review of H2S and Nitrogen-Based Contaminants from Biogas: Effects on Reforming Catalysts and SOFC Anodes

Daiana Gotardo Martinez [a], Ligia Gomes Oliveira [a,b,*], Matheus Henrique Zanardini [a], Matheus Ribeiro de Jesus Cerqueira [a], João Pedro Jenson de Oliveira [c,*], Fabio Coutinho Antunes [d], Gustavo Doubek [e], Hudson Zanin [c,*] and Julian Hunt [f]

[a] International Center for Renewable Energy and Biogas (CIBiogás), (Itaipu Technological Park), St. Presidente Tancredo Neves, 6731, 85867-900, Foz do Iguaçu, Parana, Brazil

[b] Renewable Materials and Energy Laboratory (LABMATER), Federal University of Parana (UFPR - Setor Palotina), St. Pioneiro, 2153, 85950-000, Palotina, Parana, Brazil

[c] School of Electrical and Computer Engineering, University of Campinas, Av. Albert Einstein 400, Campinas, SP 13083-852, Brazil

[d] Centre for Energy and Oil Studies, University of Campinas, Av. Cora Coralina 350, Campinas, SP 13083-896, Brazil.

[e] School of Chemical Engineering, University of Campinas, Av. Albert Einstein 500, Campinas, SP 13083-852, Brazil.

[f] Biological and Environmental Science and Engineering Division, King Abdullah University of Science and Technology, Thuwal, 23955-6900, Saudi Arabia.

*Corresponding author: gomesoliveira.ligia@gmail.com; joaojenson1@gmail.com; hzanin@unicamp.br

Table S1. Alternatives technologies for highly efficient and selective H_2S removal processes.

Technology	Removal Efficiency	Advantages	OPEX	Ref.
Biological bubble−column (BBC)	88%	Process efficiency; Lower operational costs.	Biological compounds; Disposal of solution.	[1–3]
Biotrickling filter (BTF)	100%	Capability to remove VOCs*.	Energy costs; Maintenance and repairs; pH adjusters and nutrients for maintaining optimal microbial activity.	[4–6]
Chemical absorption (Fe−EDTA*−carbonate)	96.8 – 100%	Simultaneous removal of CO_2 and oxygen (O_2) and increased CH_4.	Chemicals such as Fe−EDTA and carbonate; Implementation of recycling and regeneration systems.	[7–9]
Absorption (membrane bioreactors/ HFMB*)	99%	Simultaneous removal of NH_3.	Pumps; Perform cleaning and maintenance on membranes to prevent fouling.	[10–12]
Chemical absorption (ionic liquid solution/addition of MOFs*)	80 – 100%	Regeneration capacity.	High implementation cost with chemical reagents.	[13,14]

Adsorption (biochar)	95%	Regeneration capacity.	Uses of reagents for activation and impregnation of selective compounds for H_2S ($CaCO_3$−ZnO).	[15–17]
Adsorption (Cu/AC*)	132.22 ($mg_{H2S}.g^{-1}$)	Low production cost and regenerative capacity.	Reagent for material activation and textural analysis requirements.	[18,19]
Adsorption (ZnO−CuO/AC)	51.12 ($mg_{H2S}.g^{-1}$)	Low production cost and regenerative capacity.	Reagent for material activation and textural analysis requirements.	[20,21]
Adsorption (BBC/AC)	20.61 ($mg_{H2S}.g^{-1}$)	Low production cost and regenerative capacity.	Reagent for material activation and textural analysis requirements.	[16,22,23]
Adsorption (TiO_2/zeolite)	0.13 mmol/g	Low cost, and moderate reaction conditions; High selectivity for H_2S and regenerative capacity	Adsorbent regeneration; Maintenance and replacement, expenses for analysis and monitoring.	[24–26]

Adsorption (AgNaA nano zeolite)	33.24 (mg.g^{-1})	Ambient temperature and atmospheric pressure, regeneration capacity.	Acquisition of Ag and the need for regeneration in a short time of use.	26
Adsorption (Fresh zeolite)	94%	Easy operational conditions, high selectivity for H_2S and regenerative capacity.	Acquisition of zeolite, the need for regeneration.	27
Biological desulfurization	>99%	In−situ process (during AD); High efficiency of H_2S removal with low impacts during organic matter degradation and CH_4 production. Simple technique with low investment and operating costs;	Micro−aeration system maintenance.	28–30

Low energy demand.

***VOCs:** volatile organic compounds;
EDTA: Ethylenediaminetetraacetic acid;
MOFs: Metal−organic frameworks;
BBC: Babassu−derived biochar;
HFMB: Hollow Fiber Membrane Bioreactor;
AC: Activated carbon.

Table S2. Summary of substrates and parameters for H_2S removal efficiency using micro−aeration.

Substrate	Scale	Digester volume (m^3)	Air/O_2 dose ($m^3.h^{-1}$)	Initial H_2S content (ppmv)	H_2S removal efficiency (%)	Ref.
Sewage sludge	Full	30,000	6.00	1,889	73.8	31
		1,600	1.00	2,662	87.9	
		1,900	0.28	638	94.6	
		3,200	1.00	2,743	94.8	
		2,600	0.34	553	96.0	
		2,100	1.20	5,438	99.1	
		3,000	1.20	3,144	99.5	
Sewage sludge and segregated domestic food waste	Full	4,500	−	1,200 − 2,500	>99	32

Chicken manure	Lab–scale	–	–	10,400	58%	33
Swine manure	Full	700	–	–	98%	34
Municipal	Full	6,100	–	1,555	–	35
Swine Slaughterhouse (JBS S.A. – Ituiutaba)	Full	–	–	1,500	>90%	36
Swine manure	Lab–scale	8.5	0.12	3,500	>99%	37
Sewage sludge and cheese whey	Lab–scale	0.02	0.004	–	97%	29
High–solid sewage sludge	Full	4,500	–	100	98.2%	38
Sewage sludge	Full	2,450	4 – 14	5,500	80%	39
		3,250	5.7	1,000	80%	
		3,300	5.1	2,250	87%	
Sewage sludge	Full	0,2	0.05	3300–5,000	99%	40

Table S3. Operating conditions (catalyst type, pressure and temperature) and process efficiencies related to the conversion efficiency of CH_4/CO_2 and molar ratio of H_2/CO in the syngas, based on dry, autothermal and steam reforming.

Process	Catalyst	Pressure	T (ºC)	Conversion efficiency CH_4/CO_2 (%)	Molar Ratio H_2/CO	Ref.
DRM	Ni/M−Al_2O_3	atm	700	77.6/85.4	0.85	[41]
DRM	Ni/Si−MCM−41	atm	800	99/97	0.85−1.2	[42]
DRM	Pt/$Mg_{1-x}Ni_xO$	−	900	95/95	~1,25	[43]
DRM	Ni/SBA−15	atm	750	91/94	~1	[44]
ATR	1.5Rh/CZLa15	atm	850	75 of CH_4	−	[45]
ATR	Ni/$CaTiO_3$ and Ni/$BaTiO_3$	atm	800	70 of CH_4	−	[46]
ATR	Pt/$Ce_{0.75}Zr_{0.25}O_2$	atm	800	~75 of CH_4	~2.0	[47]
ATR	Rh/$Ce_{0.5}Zr_{0.5}O_2$	atm	850	57.62 of CH_4	3.52	[48]
SRM	Ni/$MgAl_2O_4$	2 bar	750	98 of CH_4	−	[49]
SRM	$CeO_2/La_2Ni_{1.4}Co_{.6}O_6$	−	850	86 of CH_4	2	[50]
SRM	$La_{0.95}Ce_{0.05}Ni_{0.2}Fe_{0.8}O_3$ and $La_{0.95}Ce_{0.05}Ni_{0.5}Fe_{0.5}O_3$	−	750	93.1 of CH_4 and 95.7 of CH_4	2	[51]
SRM	Sr–Ni/MCM−41	atm	700	55.81 of CH_4	3.1	[52]

SRM	Ni/γ−Al_2O_3	300 kPa	450	>80 of CH_4	3	[53]
SRM	Pd–Rh<br>Ni/Al_2O_3<br>Ru/Al_2O_3	−	800	97.2 of CH_4<br>93.7 of CH_4<br>94.9 of CH_4	5.70<br>5.5<br>6.03	[54]

References

(1) Jiang, X.; Wu, J.; Jin, Z.; Yang, S.; Shen, L. Enhancing the Removal of H2S from Biogas through Refluxing of Outlet Gas in Biological Bubble−Column. *Bioresour Technol* **2020**, *299*, 122621. https://doi.org/10.1016/j.biortech.2019.122621.

(2) Rouhollahi, A. A.; Giyahchi, M.; Dastgheib, S. M. M.; Moghimi, H. Assessing the Efficiency and Microbial Diversity of H2S−Removing Biotrickling Filters at Various PH Conditions. *Microb Cell Fact* **2024**, *23* (1), 157. https://doi.org/10.1186/s12934−024−02427−9.

(3) Rocher−Rivas, R.; González−Sánchez, A.; Ulloa−Mercado, G.; Muñoz, R.; Quijano, G. Biogas Desulfurization and Calorific Value Enhancement in Compact H2S/CO2 Absorption Units Coupled to a Photobioreactor. *J Environ Chem Eng* **2022**, *10* (5), 108336. https://doi.org/10.1016/J.JECE.2022.108336.

(4) Watsuntorn, W.; Khanongnuch, R.; Chulalaksananukul, W.; Rene, E. R.; Lens, P. N. L. Resilient Performance of an Anoxic Biotrickling Filter for Hydrogen Sulphide Removal from a Biogas Mimic: Steady, Transient State and Neural Network Evaluation. *J Clean Prod* **2020**, *249*, 119351. https://doi.org/10.1016/j.jclepro.2019.119351.

(5) Do Vale Borges, A.; Zamariolli Damianovic, M. H. R.; Torre, R. M. Assessment of Aerobic−Anoxic Biotrickling Filtration for the Desulfurization of High−Strength H2S Streams from Sugarcane Vinasse Fermentation. *J Hazard Mater* **2025**, *489*, 137696. https://doi.org/10.1016/J.JHAZMAT.2025.137696.

(6) Fortuny, M.; Baeza, J. A.; Gamisans, X.; Casas, C.; Lafuente, J.; Deshusses, M. A.; Gabriel, D. Biological Sweetening of Energy Gases Mimics in Biotrickling Filters. *Chemosphere* **2008**, *71* (1), 10–17. https://doi.org/10.1016/j.chemosphere.2007.10.072.

(7) Marín, D.; Vega, M.; Lebrero, R.; Muñoz, R. Optimization of a Chemical Scrubbing Process Based on a Fe−EDTA−Carbonate Based Solvent for the Simultaneous Removal of CO2 and H2S from Biogas. *Journal of Water Process Engineering* **2020**, *37*, 101476. https://doi.org/10.1016/j.jwpe.2020.101476.

(8) Schiavon Maia, D. C.; Niklevicz, R. R.; Arioli, R.; Frare, L. M.; Arroyo, P. A.; Gimenes, M. L.; Pereira, N. C. Removal of H 2 S and CO 2 from Biogas in Bench Scale and the Pilot Scale Using a Regenerable Fe−EDTA Solution. *Renew Energy* **2017**, *109*, 188–194. https://doi.org/10.1016/j.renene.2017.03.023.

(9) Wubs, H. J.; Beenackers, A. A. C. M. Kinetics of the Oxidation of Ferrous Chelates of EDTA and HEDTA in Aqueous Solution. *Ind Eng Chem Res* **1993**, *32* (11), 2580–2594. https://doi.org/10.1021/IE00023A022/ASSET/IE00023A022.FP.PNG_V03.
(10) Das, J.; Nolan, S.; Lens, P. N. L. Simultaneous Removal of H2S and NH3 from Raw Biogas in Hollow Fibre Membrane Bioreactors. *Environ Technol Innov* **2022**, *28*, 102777. https://doi.org/10.1016/j.eti.2022.102777.
(11) Tantikhajorngosol, P.; Laosiripojana, N.; Jiraratananon, R.; Assabumrungrat, S. Physical Absorption of CO2 and H2S from Synthetic Biogas at Elevated Pressures Using Hollow Fiber Membrane Contactors: The Effects of Henry's Constants and Gas Diffusivities. *Int J Heat Mass Transf* **2019**, *128*, 1136–1148. https://doi.org/10.1016/j.ijheatmasstransfer.2018.09.076.
(12) Brunetti, A.; Lei, L.; Avruscio, E.; Karousos, D. S.; Lindbråthen, A.; Kouvelos, E. P.; He, X.; Favvas, E. P.; Barbieri, G. Long−Term Performance of Highly Selective Carbon Hollow Fiber Membranes for Biogas Upgrading in the Presence of H2S and Water Vapor. *Chemical Engineering Journal* **2022**, *448*, 137615. https://doi.org/10.1016/j.cej.2022.137615.
(13) Li, M.; Dong, X.; Gao, P.; Yin, L.; Chen, G.; Gao, P.; Liu, X. The Crucial Role of NMOFs in H2S Absorption Process Using Ionic Liquid Solution Based Nanofluid Systems. *Chemical Engineering Journal* **2024**, *480*, 148072. https://doi.org/10.1016/j.cej.2023.148072.
(14) Zhou, Z.; Zhang, P.; Chang, Y.; Chen, X. Highly Efficient Capture and Removal of H2S by Multi−Amine Functionalized Ionic Liquids. *J Mol Liq* **2023**, *392*, 123501. https://doi.org/10.1016/J.MOLLIQ.2023.123501.
(15) Yuan, Y.; Huang, L.; Zhang, T. C.; Wang, Y.; Yuan, S. CaCO3−ZnO Loaded Scrap Rice−Derived Biochar for H2S Removal at Room−Temperature: Characterization, Performance and Mechanism. *Fuel Processing Technology* **2023**, *249*, 107846. https://doi.org/10.1016/j.fuproc.2023.107846.
(16) Scheufele, F. B.; da Silva, E. S.; Cazula, B. B.; Marins, D. S.; Sequinel, R.; Borba, C. E.; Patuzzo, G. S.; Lopez, T. F. M.; Alves, H. J. Mathematical Modeling of Low−Pressure H2S Adsorption by Babassu Biochar in Fixed Bed Column. *J Environ Chem Eng* **2021**, *9* (1), 105042. https://doi.org/10.1016/j.jece.2021.105042.
(17) Seo, D.−C.; Guo, R.; Lee, D.−H. Performance of Alkaline Impregnated Biochar Derived from Rice Hull for Hydrogen Sulfide Removal from Gas. *Environmental Engineering Research* **2020**, *26* (6), 200452–0. https://doi.org/10.4491/eer.2020.452.
(18) Wang, S.; Nam, H.; Lee, D.; Nam, H. H2S Gas Adsorption Study Using Copper Impregnated on KOH Activated Carbon from Coffee Residue for Indoor Air Purification. *J Environ Chem Eng* **2022**, *10* (6), 108797. https://doi.org/10.1016/j.jece.2022.108797.
(19) Kim, H.; Ko, K.−J.; Mofarahi, M.; Kim, K.−M.; Lee, C.−H. Adsorption Behavior and Mechanism of Ultra−Low Concentration Sulfur Compounds in Natural Gas on Cu−Impregnated Activated Carbon. *Chemical Engineering Journal* **2023**, *470*, 144274. https://doi.org/10.1016/j.cej.2023.144274.

(20) Balsamo, M.; Cimino, S.; de Falco, G.; Erto, A.; Lisi, L. ZnO−CuO Supported on Activated Carbon for H2S Removal at Room Temperature. *Chemical Engineering Journal* **2016**, *304*, 399–407. https://doi.org/10.1016/j.cej.2016.06.085.
(21) Garces, H. F.; Galindo, H. M.; Garces, L. J.; Hunt, J.; Morey, A.; Suib, S. L. Low Temperature H2S Dry−Desulfurization with Zinc Oxide. *Microporous and Mesoporous Materials* **2010**, *127* (3), 190–197. https://doi.org/10.1016/J.MICROMESO.2009.07.022.
(22) Coppola, G.; Papurello, D. Biogas Cleaning: Activated Carbon Regeneration for H2S Removal. *Clean Technologies* **2018**, *1* (1), 40–57. https://doi.org/10.3390/cleantechnol1010004.
(23) Mohamad Nor, N.; Sukri, M. F. F.; Mohamed, A. R. Development of High Porosity Structures of Activated Carbon via Microwave−Assisted Regeneration for H2S Removal. *J Environ Chem Eng* **2016**, *4* (4), 4839–4845. https://doi.org/10.1016/j.jece.2016.02.007.
(24) Liu, C.; Zhang, R.; Wei, S.; Wang, J.; Liu, Y.; Li, M.; Liu, R. Selective Removal of H 2 S from Biogas Using a Regenerable Hybrid TiO 2 /Zeolite Composite. *Fuel* **2015**, *157*, 183–190. https://doi.org/10.1016/j.fuel.2015.05.003.
(25) Liu, X.; Wang, R. Effective Removal of Hydrogen Sulfide Using 4A Molecular Sieve Zeolite Synthesized from Attapulgite. *J Hazard Mater* **2017**, *326*, 157–164. https://doi.org/10.1016/j.jhazmat.2016.12.030.
(26) Bahraminia, S.; Anbia, M.; Koohsaryan, E. Hydrogen Sulfide Removal from Biogas Using Ion−Exchanged Nanostructured NaA Zeolite for Fueling Solid Oxide Fuel Cells. *Int J Hydrogen Energy* **2020**, *45* (55), 31027–31040. https://doi.org/10.1016/j.ijhydene.2020.08.091.
(27) Pourzolfaghar, H.; Ismail, M. H. S. Study of H2S Removal Efficiency of Virgin Zeolite in POME Biogas Desulfurization at Ambient Temperature and Pressure. In *Developments in Sustainable Chemical and Bioprocess Technology*; Springer US: Boston, MA, 2013; pp 295–301. https://doi.org/10.1007/978−1−4614−6208−8_35.
(28) Díaz, I.; Ramos, I.; Fdz−Polanco, M. Economic Analysis of Microaerobic Removal of H2S from Biogas in Full−Scale Sludge Digesters. *Bioresour Technol* **2015**, *192*, 280–286. https://doi.org/10.1016/j.biortech.2015.05.048.
(29) Andreides, M.; Pokorná−Krayzelová, L.; Bartáček, J. Importance of Digester's Headspace Geometry for the Efficient H2S Removal through Microaeration; Experimental and Simulation Study. *Fuel* **2024**, *362*, 130900. https://doi.org/10.1016/j.fuel.2024.130900.
(30) Krayzelova, L.; Bartacek, J.; Kolesarova, N.; Jenicek, P. Microaeration for Hydrogen Sulfide Removal in UASB Reactor. *Bioresour Technol* **2014**, *172*, 297–302. https://doi.org/10.1016/j.biortech.2014.09.056.
(31) Jeníček, P.; Horejš, J.; Pokorná−Krayzelová, L.; Bindzar, J.; Bartáček, J. Simple Biogas Desulfurization by Microaeration – Full Scale Experience. *Anaerobe* **2017**, *46*, 41–45. https://doi.org/10.1016/j.anaerobe.2017.01.002.
(32) Giordano, A.; Di Capua, F.; Esposito, G.; Pirozzi, F. Long−Term Biogas Desulfurization under Different Microaerobic Conditions in Full−Scale Thermophilic

Digesters Co−Digesting High−Solid Sewage Sludge. *Int Biodeterior Biodegradation* **2019**, *142*, 131–136. https://doi.org/10.1016/j.ibiod.2019.05.017.
(33) Mahdy, A.; Song, Y.; Salama, A.; Qiao, W.; Dong, R. Simultaneous H2S Mitigation and Methanization Enhancement of Chicken Manure through the Introduction of the Micro−Aeration Approach. *Chemosphere* **2020**, *253*, 126687. https://doi.org/10.1016/j.chemosphere.2020.126687.
(34) Tápparo, D. C.; Cândido, D.; Steinmetz, R. L. R.; Etzkorn, C.; do Amaral, A. C.; Antes, F. G.; Kunz, A. Swine Manure Biogas Production Improvement Using Pre−Treatment Strategies: Lab−Scale Studies and Full−Scale Application. *Bioresour Technol Rep* **2021**, *15*, 100716. https://doi.org/10.1016/j.biteb.2021.100716.
(35) Forouzanmehr, F.; Le, Q. H.; Solon, K.; Maisonnave, V.; Daniel, O.; Buffiere, P.; Gillot, S.; Volcke, E. I. P. Plant−Wide Investigation of Sulfur Flows in a Water Resource Recovery Facility (WRRF). *Science of The Total Environment* **2021**, *801*, 149530. https://doi.org/10.1016/j.scitotenv.2021.149530.
(36) AWITE BIOENERGIA. *Biogas and biomethane plant of the Benas Power Group in Ottersberg*. https://www.awite.de/pt−br/awigossip−i−2024/ (accessed 2024−08−01).
(37) Huertas, J. K.; Quipuzco, L.; Hassanein, A.; Lansing, S. Comparing Hydrogen Sulfide Removal Efficiency in a Field−Scale Digester Using Microaeration and Iron Filters. *Energies (Basel)* **2020**, *13* (18), 4793. https://doi.org/10.3390/en13184793.
(38) Di Costanzo, N.; Di Capua, F.; Cesaro, A.; Carraturo, F.; Salamone, M.; Guida, M.; Esposito, G.; Giordano, A. Headspace Micro−Oxygenation as a Strategy for Efficient Biogas Desulfurization and Biomethane Generation in a Centralized Sewage Sludge Digestion Plant. *Biomass Bioenergy* **2024**, *183*, 107151. https://doi.org/10.1016/j.biombioe.2024.107151.
(39) Kraakman, N. J. R.; Diaz, I.; Fdz−Polanco, M.; Muñoz, R. Large−Scale Micro−Aerobic Digestion Studies at Municipal Water Resource Recovery Facilities for Process−Integrated Biogas Desulfurization. *Journal of Water Process Engineering* **2023**, *53*, 103643. https://doi.org/10.1016/j.jwpe.2023.103643.
(40) Ramos, I.; Fdz−Polanco, M. Microaerobic Control of Biogas Sulphide Content during Sewage Sludge Digestion by Using Biogas Production and Hydrogen Sulphide Concentration. *Chemical Engineering Journal* **2014**, *250*, 303–311. https://doi.org/10.1016/j.cej.2014.04.027.
(41) Bian, Z.; Zhong, W.; Yu, Y.; Wang, Z.; Jiang, B.; Kawi, S. Dry Reforming of Methane on Ni/Mesoporous−Al2O3 Catalysts: Effect of Calcination Temperature. *Int J Hydrogen Energy* **2021**, *46* (60), 31041–31053. https://doi.org/10.1016/j.ijhydene.2020.12.064.
(42) Oliveira, L. G.; Machado, B.; Pereira de Souza, L.; Gosch Corrêa, G. C.; Polinarski, M. A.; Cavalcanti Trevisan, S. V.; Borba, C. E.; Brackmann, R.; Alves, H. J. Dry Reforming of Biogas in a Pilot Unit: Scale−up of Catalyst Synthesis and Green Hydrogen Production. *Int J Hydrogen Energy* **2022**, *47* (84), 35608–35625. https://doi.org/10.1016/j.ijhydene.2022.08.141.
(43) Al−Doghachi, F. J.; Zainal, Z.; Saiman, M. I.; Embong, Z.; Taufiq−Yap, Y. H. Hydrogen Production from Dry−Reforming of Biogas over Pt/Mg1−XNixO Catalysts. *Energy Procedia* **2015**, *79*, 18–25. https://doi.org/10.1016/j.egypro.2015.11.460.

(44) Omoregbe, O.; Danh, H. T.; Nguyen−Huy, C.; Setiabudi, H. D.; Abidin, S. Z.; Truong, Q. D.; Vo, D.−V. N. Syngas Production from Methane Dry Reforming over Ni/SBA−15 Catalyst: Effect of Operating Parameters. *Int J Hydrogen Energy* **2017**, *42* (16), 11283–11294. https://doi.org/10.1016/j.ijhydene.2017.03.146.
(45) Cao, L.; Ni, C.; Yuan, Z.; Wang, S. Autothermal Reforming of Methane Over CeO2–ZrO2–La2O3 Supported Rh Catalyst. *Catal Letters* **2009**, *131* (3–4), 474–479. https://doi.org/10.1007/s10562−009−9888−z.
(46) Araújo, P. M.; da Costa, K. M.; Passos, F. B. Hydrogen Production from Methane Autothermal Reforming over CaTiO3, BaTiO3 and SrTiO3 Supported Nickel Catalysts. *Int J Hydrogen Energy* **2021**, *46* (47), 24107–24116. https://doi.org/10.1016/j.ijhydene.2021.04.202.
(47) Ruiz, J. A. C.; Passos, F. B.; Bueno, J. M. C.; Souza−Aguiar, E. F.; Mattos, L. V.; Noronha, F. B. Syngas Production by Autothermal Reforming of Methane on Supported Platinum Catalysts. *Appl Catal A Gen* **2008**, *334* (1–2), 259–267. https://doi.org/10.1016/j.apcata.2007.10.011.
(48) Cao, L.; Pan, L.; Ni, C.; Yuan, Z.; Wang, S. Autothermal Reforming of Methane over Rh/Ce0.5Zr0.5O2 Catalyst: Effects of the Crystal Structure of the Supports. *Fuel Processing Technology* **2010**, *91* (3), 306–312. https://doi.org/10.1016/j.fuproc.2009.11.001.
(49) Lim, T.−W.; Hwang, D.−H.; Choi, Y.−S. Design and Optimization of a Steam Methane Reformer for Ship−Based Hydrogen Production on LNG−Fueled Ship. *Appl Therm Eng* **2024**, *243*, 122588. https://doi.org/10.1016/j.applthermaleng.2024.122588.
(50) Wang, F.; Chen, S.; Chen, S.; Du, J.; Duan, L.; Xiang, W. Double Adjustment of Ni and Co in CeO2/La2Ni2−XCoxO6 Double Perovskite Type Oxygen Carriers for Chemical Looping Steam Methane Reforming. *Chemical Engineering Journal* **2023**, *465*, 143041. https://doi.org/10.1016/j.cej.2023.143041.
(51) Zhao, K.; Zhang, R.; Gao, Y.; Lin, Y.; Liu, A.; Wang, X.; Zheng, A.; Huang, Z.; Zhao, Z. High Syngas Selectivity and near Pure Hydrogen Production in Perovskite Oxygen Carriers for Chemical Looping Steam Methane Reforming. *Fuel Processing Technology* **2022**, *236*, 107398. https://doi.org/10.1016/j.fuproc.2022.107398.
(52) Hasani Estalkhi, M.; Yousefpour, M.; Koohestan, H.; Taherian, Z. Catalytic Evaluation of Ni–3%Sr−/MCM−41 in Dry and Steam Reforming of Methane. *Int J Hydrogen Energy* **2024**, *68*, 1344–1351. https://doi.org/10.1016/j.ijhydene.2024.04.347.
(53) Ighalo, J. O.; Amama, P. B. Recent Advances in the Catalysis of Steam Reforming of Methane (SRM). *Int J Hydrogen Energy* **2024**, *51*, 688–700. https://doi.org/10.1016/j.ijhydene.2023.10.177.
(54) Roy, P. S.; Park, N.−K.; Kim, K. Metal Foam−Supported Pd–Rh Catalyst for Steam Methane Reforming and Its Application to SOFC Fuel Processing. *Int J Hydrogen Energy* **2014**, *39* (9), 4299–4310. https://doi.org/10.1016/j.ijhydene.2014.01.004.
